# Multi‐target attention and visual short‐term memory capacity are closely linked in the intraparietal sulcus

**DOI:** 10.1002/hbm.24618

**Published:** 2019-05-06

**Authors:** Maren Praß, Bianca de Haan

**Affiliations:** ^1^ Division of Neuropsychology, Center of Neurology Hertie‐Institute for Clinical Brain Research, University of Tübingen Tübingen Germany; ^2^ Division of Psychology, Department of Life Sciences College of Health and Life Sciences, Brunel University London Uxbridge UK

**Keywords:** attention, functional magnetic resonance imaging, healthy volunteers, parietal lobe, repetitive transcranial magnetic stimulation, working memory

## Abstract

The existing literature suggests a critical role for both the right intraparietal sulcus (IPS) and the right temporo‐parietal junction (TPJ) in our ability to attend to multiple simultaneously‐presented lateralized targets (multi‐target attention), and the failure of this ability in extinction patients. Currently, however, the precise role of each of these areas in multi‐target attention is unclear. In this study, we combined the theory of visual attention (TVA) with functional magnetic resonance imaging (fMRI) guided continuous theta burst stimulation (cTBS) in neurologically healthy subjects to directly investigate the role of the right IPS and TPJ in multi‐target attention. Our results show that cTBS at an area of the right IPS associated with multi‐target attention elicits a reduction of visual short‐term memory capacity. This suggests that the right IPS is associated with a general capacity‐limited encoding mechanism that is engaged regardless of whether targets have to be attended or remembered. Curiously, however, cTBS to the right IPS failed to elicit extinction‐like behavior in our study, supporting previous suggestions that different areas of the right IPS may provide different contributions to multi‐target attention. CTBS to the right TPJ failed to induce a change in either TVA parameters or extinction‐like behavior.

## 1 INTRODUCTION

Multi‐target attention, that is, the ability to attend to multiple visual targets presented simultaneously across both visual fields, is essential for everyday real‐world behavior such as navigating traffic scenes, engaging in team sports, and playing a videogame. The importance of this ability is demonstrated impressively in neurological patients suffering from extinction, most commonly as a consequence of right hemispheric brain damage (Becker & Karnath, 2007). These patients are able to report single (unilateral) visual targets in either visual field, but fail to report the contralesional target in (bilateral) situations where an ipsilesional target is concurrently present (de Haan, Karnath, & Driver, 2012; Oppenheim, 1885). An explanation for this failure to report contralesional targets during bilateral stimulation in extinction patients is given by the biased competition model (Desimone, 1998; Desimone & Duncan, 1995; Duncan, 1998; Duncan, Humphreys, & Ward, 1997). This model proposes that objects in the visual scene compete for access to limited attentional resources. This competition can be biased toward objects that are more salient (bottom‐up bias) or behaviorally more relevant (top‐down bias) than other objects. According to this model, the unilateral brain damage in extinction patients elicits a spatio‐attentional bias against objects presented in the contralesional visual field. As such, a target in the contralesional hemifield is less likely to “survive” the competition for attentional selection if another target is simultaneously presented in the ipsilesional hemifield. This lateralized spatio‐attentional deficit in extinction patients may be exacerbated by nonlateralized deficits of selective attention, such as a reduction of attentional capacity, so that ultimately only the ipsilesional target is attentionally selected and available for overt report (de Haan et al., 2012; Driver, Mattingley, Rorden, & Davis, 1997; Karnath, 1988). This latter argument is supported by the observation that the presence of a spatio‐attentional bias against contralesional targets is not specific to extinction patients (de Haan, Stoll, et al., 2015) and the observation that instructing extinction patients to report the contralesional item first during bilateral trials results in extinction of the ipsilesional target (Karnath, 1988).

Two brain regions are thought to be involved in our ability to attend to multiple simultaneously‐presented lateralized targets, and the failure of this ability in extinction patients. On the one hand, patient studies suggest that extinction is critically associated with damage to or hypoperfusion of the right (Grandjean, Sander, Lucas, Scherer, & Vuilleumier, 2008; Karnath, Himmelbach, & Kuker, 2003; Ticini, de Haan, Klose, Nagele, & Karnath, 2010), or left and right (Chechlacz et al., 2013) temporo‐parietal junction (TPJ). Moreover, in one transcranial magnetic stimulation (TMS) study, extinction‐like behavior was induced by transient neuroinhibition of the right TPJ in healthy subjects (Meister et al., 2006), supporting these results from patient studies. On the other hand, several studies (additionally) suggest a role for the intraparietal sulcus (IPS) in multi‐target attention. In neurologically healthy subjects, the right (de Haan, Bither, et al., 2015), or right and left (Çiçek, Gitelman, Hurley, Nobre, & Mesulam, 2007; Geng et al., 2006) IPS show(s) higher levels of neural activation when attention is bilaterally oriented compared to situations where the focus of attention is unilateral. Moreover, several TMS studies found extinction‐like behavior in healthy subjects after a transient inhibition of neural activity at the right (Cazzoli, Müri, Hess, & Nyffeler, 2009), or left and right (Battelli, Alvarez, Carlson, & Pascual‐Leone, 2009; Hilgetag, Théoret, & Pascual‐Leone, 2001; Pascual‐Leone et al., 1994) IPS. Thus, the existing literature suggests that both the IPS and the TPJ might be important for our ability to attend to multiple simultaneously‐presented lateralized targets. Moreover, in line with the view that the right hemisphere is dominant for visuospatial attention (Heilman & Van den Abell, 1980; Kinsbourne, 1993; Mesulam, 1981), the literature suggests that the right IPS and TPJ may be particularly important for this ability. Currently, however, the precise contribution of each of these two areas to multi‐target attention is unknown.

The theory of visual attention (TVA; Bundesen, 1990, 1998) is ideally placed to clarify the precise contribution of the right IPS and TPJ to our ability to attend to multiple simultaneously‐presented lateralized targets (and the failure of this ability in extinction patients). TVA is a mathematical model of visual attention, closely related to the biased competition model. According to TVA, attentional selection is not a unitary process, but consists of different attentional sub‐processes that can each be individually measured. In brief (see Bundesen & Habekost, 2008 for a more detailed explanation), TVA assumes that performance in visual attention tasks is determined by four parameters: (a) processing speed (*C*), indicating the rate at which stimuli can be processed, (b) visual short‐term memory (VSTM) capacity (*K*), indicating the maximum amount of stimuli that can be simultaneously represented in VSTM, (c) spatial distribution of attention (*w*_*λ*_), indicating the direction of the overall spatio‐attentional bias, and (d) top‐down control (*α*), indicating the attentional preference given to targets over distractors. According to TVA, visual objects are processed in parallel and compete for selection. This selection process is determined by two factors: the processing rate and VSTM capacity (*K*). Objects with a higher processing rate have a higher probability of winning the race to be encoded in VSTM. The processing speed (*C*) equals the sum of the processing rates for all objects in the visual field. The processing rate for each individual object reflects the proportion of the total processing capacity allocated to that object (i.e., its attentional weight), which depends on top‐down control (*α*) and the direction of the overall spatio‐attentional bias (*w*_*λ*_). The first *K* elements that enter VSTM will be available for overt report and object processing is terminated when the VSTM capacity limit is reached (usually 3–4 items). The optimal values of these four parameters can be estimated by fitting the TVA model to the data of two simple tasks, the whole report and the partial report of briefly presented letters. The processing speed and VSTM capacity parameters are assessed with the whole report paradigm, where subjects are presented with multiple letters and have to report as many letters as possible. The spatial distribution of attention and top‐down control parameters are assessed with the partial report paradigm. Here, subjects report only target letters, while ignoring distractor letters. The four parameters are quantitatively estimated using a trial‐by‐trial maximum likelihood fitting procedure (Dyrholm, Kyllingsbæk, Espeseth, & Bundesen, 2011).

Combining TVA with transient neuroinhibition or lesion‐behavior mapping is thus uniquely capable of providing detailed and valuable information concerning the precise attentional sub‐processes impacted by a functional impairment of the IPS or TPJ, as well as their relation to multi‐target attention. Moreover, TVA only requires participants to perform two short tasks (each taking approximately 15 min to complete) and is thus very easy to implement. Surprisingly, however, given this ease of implementation and informativeness, applications of TVA to study the precise contribution of the IPS and TPJ to attention in multi‐target environments are rare. A few studies have, however, applied TVA to assess the contribution of the IPS to selective attention. Hung, Driver, and Walsh (2005) combined TVA with on‐line repetitive transcranial magnetic stimulation (rTMS) to transiently inhibit neural processing at either the left or right posterior parietal cortex (PPC). Their results indicated that a transient inhibition of neural activity at the right PPC elicited reduced top‐down control in the contralateral visual field (higher parameter *α* after rTMS over right PPC compared to control), paired with increased top‐down control in the ipsilateral visual field. Similarly, Moos, Vossel, Weidner, Sparing, and Fink (2012) combined TVA with transcranial direct current stimulation (tDCS) to modulate neural processing at the right IPS. They, however, found that 2 mA cathodal tDCS at the right IPS elicited enhanced top‐down control in both visual fields. Together, these studies suggest that the posterior parietal cortex contributes to the ability to attentionally prioritise relevant information over irrelevant information, even if different areas within the posterior parietal cortex might play subtly different roles in this ability. Both studies, however, only used partial report, and thus did not assess the TVA parameters processing speed and VSTM capacity. Moreover, while Hung et al. (2005) interpret their results as evidence of extinction‐like behavior, it is unclear how either their, or Moos et al.'s (2012) results relate to the “classical” behavior expressed by extinction patients. As such, the precise contribution of the IPS to our ability to attend to multiple simultaneously‐presented lateralized targets remains to be clarified. Other studies have applied TVA to investigate impaired performance in visual attention tasks in stroke patients. These studies suggest that, compared to healthy subjects, chronic stroke patients with damage centering on the right inferior parietal cortex are abnormally slow to process information, show a pathological reduction of VSTM capacity and display an abnormal spatio‐attentional bias toward the ipsilesional visual field (Duncan et al., 1999). Additionally, the slowing of information processing and reduction of VSTM capacity tended to be particularly pronounced in patients with damage centering on the right TPJ (Peers et al., 2005). These studies, however, did not assess whether these deficits were associated with extinction. The only study that attempted to assess the TVA parameters affected by extinction after stroke (Habekost & Rostrup, 2006) only assessed right hemispheric chronic stroke patients with minor or no clinical signs of extinction. Consequently, the result from this study, suggesting that extinction severity correlated moderately with reduced contralesional processing speed, is difficult to interpret. Thus, while these seminal studies using TVA are promising starting points, it is clear that more research needs to be done to clarify the precise attentional sub‐processes affected after a functional impairment of either the IPS or the TPJ and their relation to our ability to attend to multiple simultaneously‐presented lateralized targets.

The current study thus aims to elucidate the role of the right IPS and the right TPJ in multi‐target attention and its failure in extinction patients. We combine TVA with functional magnetic resonance imaging (fMRI) guided continuous theta burst transcranial magnetic stimulation (cTBS) to transiently inhibit neural activation at either the right IPS or the right TPJ in neurologically healthy participants. Specifically, we use fMRI to individually define the location of the right IPS and right TPJ area associated with multi‐target attention. Subsequently, we assess the effect of cTBS‐induced transient neuroinhibition at these brain areas on the four TVA parameters and subjects' ability to attend to multiple simultaneously‐presented lateralized targets. Given the scarcity of previous studies using TVA to study the role of the IPS and TPJ in multi‐target attention, deriving precise study predictions is difficult. Nevertheless, based on the current view of extinction as a consequence of both lateralized and nonlateralized deficits, and the results of the studies presented above, we offer the following tentative hypotheses: Firstly, we hypothesize that cTBS to the right IPS will elicit a lateralized reduction of top‐down control, as well as, potentially, a reduction of processing speed and/or VSTM capacity. Secondly, we hypothesize that cTBS to the right TPJ will elicit a spatio‐attentional bias toward the ipsilateral visual field, as well as a reduction in processing speed and VSTM capacity. Finally, we hypothesize that the modulation of these attentional sub‐processes will be accompanied by a reduction in our subjects' ability to attend to multiple simultaneously‐presented lateralized targets.

## 2 MATERIALS AND METHODS

Each subject performed five experimental sessions (taking place on separate days). In the first session, subjects participated in a fMRI study (fMRI localizer), allowing us to determine the location of the right IPS and right TPJ area associated with multi‐target attention in each individual subject. In the second session, we applied cTBS at the hand area of the right motor cortex and measured the size of the motor evoked potential (MEP) in each individual subject, allowing us to qualify the effect of cTBS on cortical functioning in our subjects. Finally, in sessions 3–5, subjects participated in a TVA experiment while we applied cTBS at either the right IPS, the right TPJ, or the vertex (as a control location), allowing us to assess the effect of transient neuroinhibition at the IPS and TPJ area associated with multi‐target attention on attentional sub‐processes and our ability to attend to multiple simultaneously‐presented lateralized targets.

### 2.1 Participants

Forty subjects (19 females, mean age 25.2 years, range 18–36 years) participated in the current study. A single subject had to be excluded from the analyses due to failure to comply with the task instruction to fixate on the central fixation cross. Furthermore, four subjects were excluded from further analysis due to insufficient TVA parameter fits (see Section 3). Thus, 35 subjects (16 females, mean age 25.7 years, range 18–36 years) were available for the entire set of analyses. All subjects were healthy with no history of neurological or psychiatric disorders, were right‐handed, and had normal or corrected to normal vision. All subjects were volunteers and signed an informed consent approved by the ethics committee of the Medical Faculty of Tübingen (project number 261/2010BO1). Subjects were paid 10€ per hour for their participation.

### 2.2 Session 1: fMRI localizer

**Figure 1:**
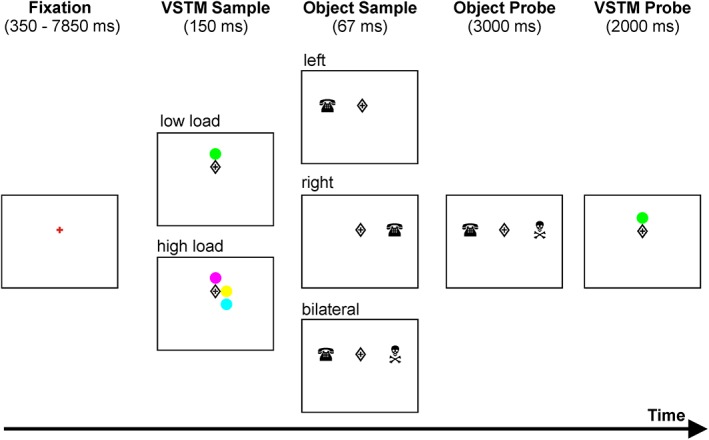
Combined VSTM and object identification task (adapted from Emrich et al., 2011). Each trial started with a fixation cross, which was followed by a VSTM sample display consisting of either 1 (low VSTM load) or 3 (high VSTM load) colored disks. Subjects were instructed to maintain both location and color of the disc(s) presented in VSTM. Following the VSTM sample display, subjects were presented with an object sample display containing either a single object in the left or the right visual field or two objects, one in each visual field. This object sample display was immediately followed by an object probe display containing one object in each visual field and subjects were instructed to indicate which object(s) from the object probe display matched the object(s) shown in the object sample display (left, right, both or none). Finally, following the object probe display, subjects were presented with a VSTM probe display containing one colored disc and instructed to indicate whether or not the color and position of the colored disc in the VSTM probe display matched the color and position of (one of) the colored disc(s) presented in the VSTM sample display displayed at the start of the trial. Thus, the object identification task was performed either under conditions of low VSTM load, or under conditions of high VSTM load. VSTM, visual short‐term memory [Color figure can be viewed at http://wileyonlinelibrary.com]

We used a rapid event‐related fMRI localizer to functionally define the location of the right IPS and right TPJ area associated with multi‐target attention in each individual subject. While the fMRI signal was continuously measured, subjects performed a combined visual short‐term memory (VSTM) and object identification task based on the task used by Emrich, Burianova, and Ferber (2011); see also Figure 1). In this task, subjects were asked to identify bilaterally or unilaterally presented objects, while simultaneously maintaining either one (low VSTM load) or three (high VSTM load) colored disks in VSTM. Thus, the object identification task was performed under either low or high VSTM maintenance load. The resulting object presentation conditions were: bilateral‐object_low‐load, left‐object_low‐load, right‐object_low‐load, bilateral‐object_high‐load, left‐object_high‐load, and right‐object_high‐load.

The choice for this task was motivated by two sets of observations from the previous literature: Firstly, previous studies have demonstrated that comparing neural activation during bilateral object identification trials and neural activation during unilateral object identification trials enables isolating the right IPS area associated with attention in multi‐target environments (Çiçek et al., 2007; de Haan, Bither, et al., 2015; Geng et al., 2006). Secondly, previous studies have demonstrated that a reduction of VSTM capacity by a concurrent VSTM maintenance task elicits a functional deactivation of the right TPJ which in turn elicits target detection deficits (Todd, Fougnie, & Marois, 2005) particularly for targets presented in the left visual field and in multi‐target environments (Emrich et al., 2011). This suggests that comparing neural activation during high VSTM load trials and neural activation during low VSTM load trials might enable isolating the right TPJ area associated with attention in multi‐target environments.

Functional imaging was performed using a 3T Siemens Magnetom Trio scanner (Erlangen, Germany). The fMRI volumes were collected axially with a flip angle of 90°, a time to echo (TE) of 40 ms and a time to repetition (TR) of 2,680 ms. Each fMRI volume contained 33 slices acquired in sequential ascending order with 3 mm^3^ voxel size without gap between slices (field of view [FOV]: 192 × 192). Additionally, for each subject we sagitally acquired a T1‐weighted Magnetization Prepared Rapid Gradient Echo (MP‐RAGE) anatomical volume (176 slices, 1 × 1 × 1 mm, 240 × 256 FOV) with a flip‐angle of 15°, a TE of 3.4 ms and a TR of 2,000 ms to aid normalization and visualization of the functional data. Preprocessing and statistical analyses were performed with SPM8 (Wellcome Department of Imaging Neuroscience, London, UK) implemented in Matlab 2014a (Mathworks, Inc., Natick, MA). The functional volumes were slice time corrected and motion corrected. Subsequently, the T1‐weighted volume was coregistered with the mean functional volume obtained after realignment. Transforms for warping the coregistered T1‐weighted volume to standard stereotaxic space were computed using the unified segmentation and normalization approach. The resulting transformation parameters were used to warp the functional volumes and structural volumes into stereotaxic space. Finally, the functional volumes were spatially smoothed (8 mm FWHM). Each object presentation condition was modeled using the standard SPM8 hemodynamic response function with temporal and dispersion derivatives. To reduce global noise, the time series of the mean white matter signal was added as a regressor.

In each individual subject, we defined the location of the right IPS area associated with multi‐target attention by performing a conjunction analysis comparing the neural activity elicited by bilateral object sample displays to the neural activation elicited by unilateral object sample displays ([(bilateral > unilateral left) AND (bilateral > unilateral right)], analogously to the approach taken in de Haan, Bither, et al. (2015)), averaged over both VSTM loads. The location of the right TPJ area associated with multi‐target attention was defined by subtracting the neural activation elicited by object sample displays presented during low VSTM load from the neural activity elicited by object sample displays presented during high VSTM load, averaged over both unilateral and bilateral object presentation conditions. Finally, the coordinates of the functionally defined IPS and TPJ were transformed from stereotaxic MNI space to native space using the deformation field image created in the normalization of the T1‐weighted volume. Full methodological details of this fMRI localizer session can be found in the Supporting Information.

### 2.3 Session 2: Effect of cTBS on MEPs

Forty seconds of cTBS has been shown to reliably induce inhibition of neural activity lasting approximately 30 min in neurologically healthy subjects (Franca, Koch, Mochizuki, Huang, & Rothwell, 2006; Hoogendam, Ramakers, & Di Lazzaro, 2010; Huang, Edwards, Rounis, Bhatia, & Rothwell, 2005), thereby offering effects that are both more consistent and of longer duration with identical or less TMS pulses applied compared to other offline TMS paradigms such as 10–15 min of 1 Hz stimulation (Hoogendam et al., 2010). Moreover, compared to online TMS paradigms (where TMS is applied while the subject performs the task), offline TMS paradigms offer the benefit that task performance is not disrupted by nonspecific effects of TMS such as discomfort, muscle twitches and auditory noise. Thus, theoretically, cTBS is ideally suited to elicit consistent long‐lasting neuroinhibition with minimal side effects. Recently, however, the consistency of this inhibitory effect of cTBS in healthy subjects has been challenged (Hamada, Murase, Hasan, Balaratnam, & Rothwell, 2013; McAllister et al., 2013). Hamada et al. (2013) found that an inhibitory effect of cTBS was present in only 42% of their subjects, while the remaining 58% of their subjects showed a facilitatory effect after cTBS. Likewise, McAllister et al. (2013) found that an inhibitory effect of cTBS was only present in 50% of their subjects, while the remaining 50% of their subjects showed either no effect, or a facilitatory effect after cTBS. Thus, there appears to be considerable interindividual variability in the effect of cTBS on cortical functioning, something that might have to be taken into account when using cTBS for neuroinhibition of cortical areas. Currently, the precise cause of this interindividual variability is unclear, but several factors have been proposed (see Ridding & Ziemann, 2010 for a review). For cTBS, this interindividual variability may be related to genetic differences between subjects (Cheeran et al., 2008; Jannati, Block, Oberman, Rotenberg, & Pascual‐Leone, 2017; Mix, Benali, & Funke, 2014). Specifically, the results from several studies suggest that the interindividual variability in the effect of cTBS on cortical functioning may be associated with the Val66Met polymorphism of the brain‐derived neurotrophic factor gene (BDNF), with neuroinhibitory after‐effects of cTBS reduced or absent in Val66Met carriers (Cheeran et al., 2008; Jannati et al., 2017). This suggests that there is a qualitative difference between subjects that show an inhibitory effect following cTBS and subjects that do not.

We thus aimed to obtain a qualitative measurement of the effect of cTBS on cortical functioning in each individual subject. For this, we applied cTBS at the hand area of the right motor cortex and measured the peak‐to‐peak amplitude of the resting motor evoked potential (MEP) in the first dorsal interosseous muscle before and after 40 s of cTBS with the aid of an electromyogram (BrainVision, BRAINAMP MR series, Brain Products GmbH, Gilching, Germany). CTBS was applied with a Magstim Superrapid TMS stimulator with integrated two channel muscle evoked potentials (MEP) recording and a 70 mm figure‐of‐eight TMS coil (The Magstim Company Ltd., Whitland, UK). The positioning of the TMS coil was guided with an optical real‐time navigation system based on a structural MRI scan in each individual subject. The navigation system consisted of a Polaris Position Sensor (Northern Digital Inc., Waterloo, ON, Canada) and the Localite TMS Navigator Software Package (Localite GmbH, Sankt Augustin, Germany). For MEP data analysis we used the BrainVision‐Analyser (Version 2.0.4.368, Brain Products GmbH, Gilching, Germany) and Matlab R2014a (Mathworks, Inc., Natick, MA).

For cTBS we used the standard protocol as described by Huang et al. (2005) consisting of three pulses at a frequency of 50 Hz that were repeated at a frequency of 5 Hz for a duration of 40 s (thus 600 pulses in total) at 70% of the resting motor threshold (i.e., the single pulse TMS intensity required to elicit a MEP with an amplitude of ~ 1 mV). Following established protocols (Hamada et al., 2013; Huang et al., 2005; McAllister et al., 2013), we estimated a pre‐cTBS interval (baseline) and seven post‐cTBS intervals. The post‐cTBS intervals were recorded every 5 min over a period of 30 min. In each interval, 30 single pulses of TMS were administered at the hand area of the right motor cortex every 4–5 s. The average MEP of each interval was estimated by calculating the peak‐to‐peak amplitude of MEP after each single pulse and then averaging these across all trials of the interval. The post‐cTBS amplitudes of each interval were then normalized to the averaged pre‐cTBS MEP. The overall cTBS effect was subsequently assessed by calculating the grand average of the normalized post‐cTBS intervals over a period of 20 min (corresponding to the duration of the TVA experiment described below). Finally, again following established protocols (Hamada et al., 2013; McAllister et al., 2013), we divided our subjects in two groups: subjects with a normalized grand average MEP below 1 were classified as “responders” and assigned to the inhibiting group (INH), and subject with a grand average MEP above 1 were classified as “nonresponders” and assigned to the noninhibiting group (NON‐INH).

### 2.4 Sessions 3–5: Effect of cTBS on performance in the TVA experiment

Each subject participated in three cTBS sessions. In each session, we used the same standard cTBS protocol as described for Session 2 (“Effect of cTBS on MEPs”). CTBS was administered at three different cortical locations, with each location being targeted in a separate session taking place on a separate day, and at least 3 days between sessions to avoid potential carry‐over effects. Two of the three cortical locations, the right IPS and the right TPJ, were functionally defined on the basis of the individual results from the functional localizer, as described for Session 1 (“fMRI localizer”). The third location was the vertex, which was anatomically defined in each individual (the highest point on the subject's head). This location served to control for potential general effects of cTBS on task performance. The order in which cTBS was applied at the different cortical locations was randomized over subjects.

The application of cTBS and the positioning of the TMS coil was identical to that described for Session 2 (“Effect of cTBS on MEPs”). To ensure that subjects maintained stable gaze fixation throughout the task we monitored eye fixation position with the aid of an electrooculogram (EOG, BrainVision, BRAINAMP MR series, Brain Products GmbH, Gilching, Germany) and BrainVision‐Recorder (Version 2.0.4.368, Brain Products GmbH, Gilching, Germany). Horizontal electrodes (left and right of the eyes) were used for saccade‐detection and vertical electrodes (above and below left eye) were used for blink control. For EOG data analysis we used the BrainVision‐Analyser (Version 2.0.4.368, Brain Products GmbH, Gilching, Germany) and Matlab R2014a (Mathworks, Inc., Natick, MA).

**Figure 2:**
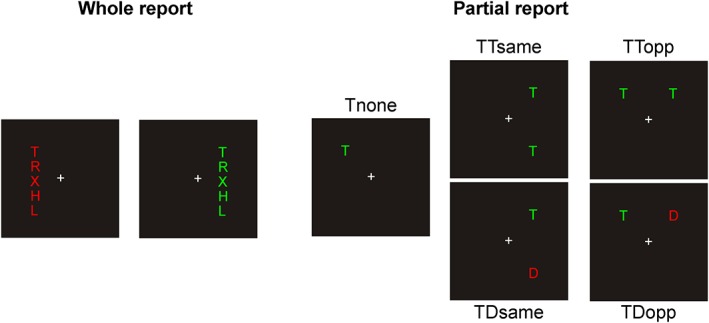
TVA tasks: whole and partial report. In the whole report task, subjects were instructed to report as many letters as they were fairly certain to have seen. In the partial report task, subjects were instructed to report only the target letters (depicted here as “T) and ignore the distractor letters (depicted here as “D”). TVA, theory of visual attention [Color figure can be viewed at http://wileyonlinelibrary.com]

In each session, subjects performed the TVA experiment, consisting of both a whole report and partial report TVA task (see Figure 2) following the application of cTBS. This allowed the estimation of all four TVA parameters. Both TVA tasks were based on the tasks used by Finke et al. (2005). In both tasks, each trial started with a 1,200 ms fixation cross (size 0.5° visual angle), centrally presented on a black background. In the whole report task, five colored letters (either all red or all green) were briefly presented to the left or right of a fixation cross, immediately followed by a pattern mask for 500 ms. Letters and masking stimuli were presented at an eccentricity of 5° visual angle and each had a size of 0.75° visual angle with a letter‐to‐letter distance of 2° visual angle. Stimulus duration varied between 10, 20, 50, 80, 140, and 200 ms. Subjects were asked to report as many letters as they were fairly certain to remember and to manually enter them in random order into a response field with their right hand. There were no time constraints for reporting the letters. Each of the six stimulus exposure durations was presented 32 times, leading to 192 trials in total. With the whole report task the parameters VSTM capacity (*K*) and processing speed (*C*) were estimated. In the partial report task, the trial sequence was exactly the same, except that either 1, or 2 colored letters were presented (red or green), followed by a pattern mask. Letters and mask subtended 0.75° × 0.75° visual angle and were presented at the corners of an imaginary square of 10° × 10° visual angle. The distance of the letters to the central fixation cross was ~7° visual angle. The two‐letter conditions contained two letters of the same color (e.g., both red) or different colors (one red, one green) and these letters were presented either vertically in the same visual field or horizontally with one letter in each visual field. Subjects were instructed to manually enter only the target letters (e.g., red letters) and ignore the distracter letters (e.g., green letters). The color of the target and distractor letters was randomly determined for each subject and session. Taken together, five possible stimulus configurations were obtained in the partial report task: 1 target letter alone (Tnone), 2 target letters in the same hemifield (TTsame), 2 target letters in opposite hemifields (TTopp), 1 target letter and 1 distractor letter in the same hemifield (TDsame), and 1 target letter and 1 distractor letter in opposite hemifields (TDopp). In total, 16 different conditions were obtained (4 single target, 8 target‐distractor, and 4 target‐target), and each condition was presented 18 times (leading to a total trial number of 288). Prior to the partial report task, a pretest was used to estimate the individual presentation duration of the stimuli in the main task. This pretest contained 32 Tnone trials where each single letter was presented for a duration of 70 ms. For subjects that performed within a mean accuracy of 60%–80%, stimulus exposure duration was kept at 70 ms in the main experiment. For subjects whose mean accuracy was outside of this range, the exposure duration was adjusted as follows: performance <50%, exposure duration extended to 100 ms; performance between 50 and 60%, exposure duration extended to 86 ms; performance between 80 and 90%, exposure duration shortened to 57 ms; performance above 90%, exposure duration shortened to 43 ms. With the partial report task the parameters top‐down selectivity (*α*) for the left and right hemifield and spatial distribution of attention (*w*_*λ*_) were estimated, where the parameter *w*_*λ*_ is defined as the ratio of left and right target and distractor weights (*w*_*L*_/[*w*_*L*_ + *w*_*R*_]). In both tasks, responses were collected with a keyboard and performance accuracy was recorded. The order of the whole report and partial report tasks was randomized over subjects and sessions and during the first TVA session subjects performed several practice trials to familiarize themselves with the tasks.

The behavioral data from the TVA experiment was analysed in SPSS (Version 20, IBM SPSS Statistics, Armonk, NY). As outlined in the introduction, our main aim was to clarify the precise attentional sub‐processes affected after a functional impairment of either the IPS or the TPJ and their relation to our ability to attend to multiple simultaneously‐presented lateralized targets. Thus, as we were primarily interested in assessing the effect of neuroinhibition at either the IPS or the TPJ, and not in directly comparing the effects of neuroinhibition between the IPS and the TPJ, we separately compared TVA parameter estimates following cTBS at the right IPS and TVA parameter estimates following cTBS at the right TPJ with TVA parameter estimates following cTBS to the vertex. Importantly, we did a‐priorily not necessarily expect neuroinhibition at the IPS and the TPJ to differentially modulate either individual TVA parameter estimates or extinction‐like behavior. In fact, based on the current view of extinction as a consequence of both lateralized and nonlateralized deficits, and the results of previous studies, we hypothesized that several TVA parameter estimates (e.g., processing speed, VSTM capacity), as well as extinction‐like behavior, might be similarly affected following cTBS to the IPS and the TPJ. Thus, directly comparing the effects of neuroinhibition between the IPS and TPJ would potentially have obscured effects that we were interested in.

Additionally, from the partial report task, we used the proportion correct (ranging from 0 to 1) during single target (Tnone) presentations in left (contralateral) and right (ipsilateral) visual field, and double target presentations in opposite hemifields (TTopp) to calculate an extinction index according to the following formula *I*_ext_ = (*P*_(hit│uni‐left)_ − *P*_(hit│bil‐left)_)–(*P*_(hit│uni‐right)_ − *P*_(hit│bil‐right)_; taken from Vossel et al., 2011). This extinction index ranges from 1 to −1 with a score of 1 reflecting complete contralateral extinction and a score of −1 reflecting complete ipsilateral extinction. This extinction index was subsequently used to separately compare multi‐target attention following cTBS to the right IPS and multi‐target attention following cTBS at the right TPJ with multi‐target attention following cTBS to the vertex. TVA parameters were estimated using the Matlab toolbox LIBTVA (Dyrholm et al., 2011). Reported *p*‐values were Bonferroni corrected for multiple comparisons when appropriate, in which case the corrected *p*‐values are additionally noted as *p*_corr_.

## 3 RESULTS

### 3.1 fMRI localizer

**Figure 3:**
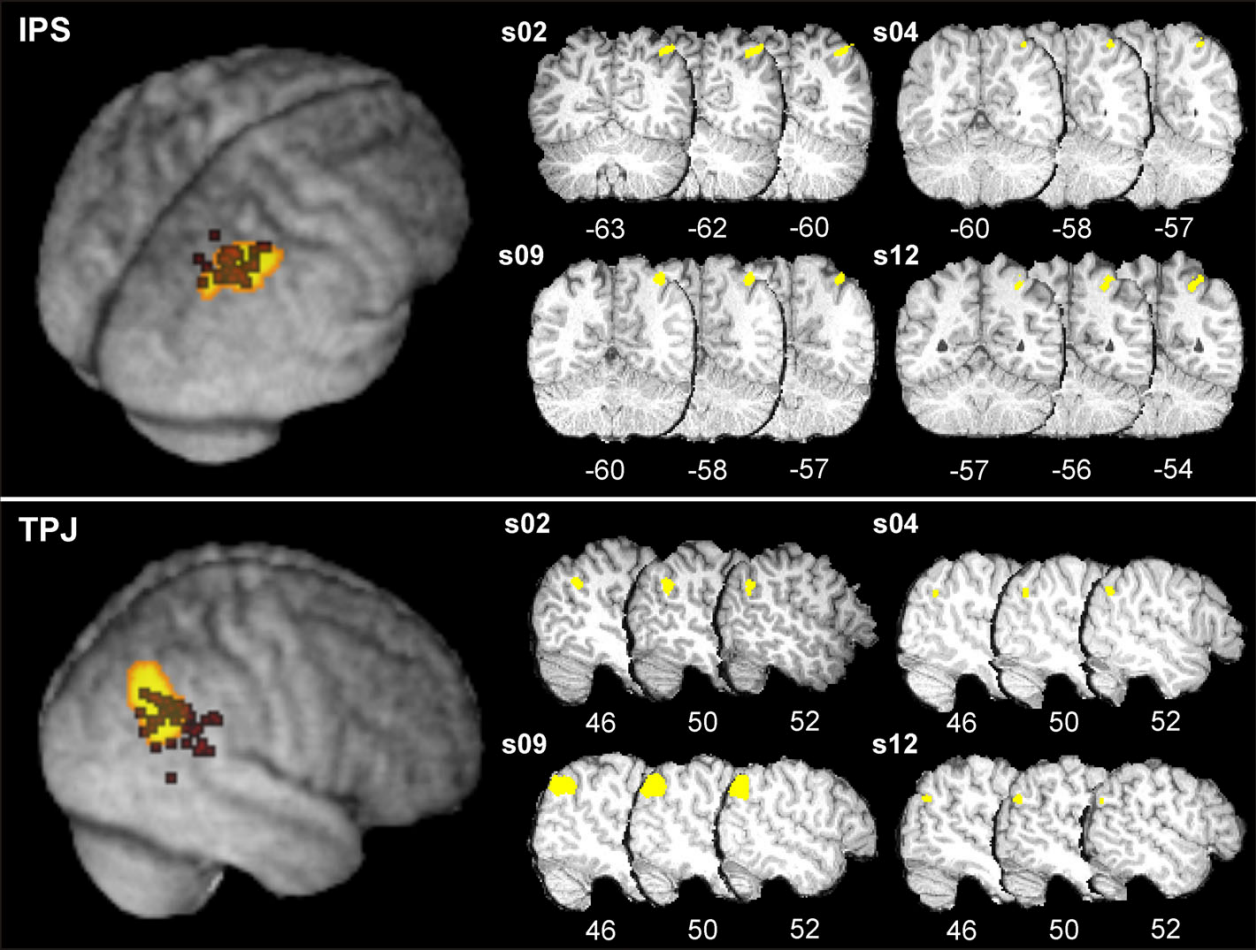
Group activation map and individual cTBS application coordinates (left side), as well as the individual results of four representative subjects (right side) for both the right IPS (upper row) and the right TPJ (lower row). All images are in neurological orientation and slice numbers reflect MNI coordinates. IPS, intraparietal sulcus; TPJ, temporo‐parietal junction [Color figure can be viewed at http://wileyonlinelibrary.com]

The aim of the fMRI localizer session was to functionally define the location of the right IPS and the right TPJ area associated with multi‐target attention in each individual subject. The location of the IPS was determined with the aid of a conjunction analysis, where we assessed where in the right posterior parietal cortex [(bilateral > unilateral left) AND (bilateral > unilateral right)] held, that is, higher neural activity during bilateral object presentation than during both unilateral left and unilateral right object presentation. The top row of Figure 3 depicts the average group cluster of the right IPS (*p* < .05 FWE corrected for multiple comparisons) and the individual peak coordinates of all subjects (left side), as well as the activation cluster in four representative subjects (right side). In two subjects the IPS coordinates could not be estimated with the conjunction contrast. Instead, we used the group peak coordinate in one subject, and another (more liberal) contrast in the other subject (contrast: 2*bilateral > [unilateral left + unilateral right]) to define the location of the IPS. The location of the TPJ was determined by assessing where in the inferior parietal lobe/superior temporal gyrus neural activation was lower during object presentation under high VSTM load than during object presentation under low VSTM load (contrast: low > high). The bottom row of Figure 3 depicts the average group cluster of the right TPJ (*p* < .05 FWE corrected for multiple comparisons) and the individual peak coordinates of all subjects (left side), as well as the activation cluster in four representative subjects (right side). The behavioral results of this fMRI localizer session can be found in the Supporting Information.

### 3.2 Effect of cTBS on MEPs

**Figure 4:**
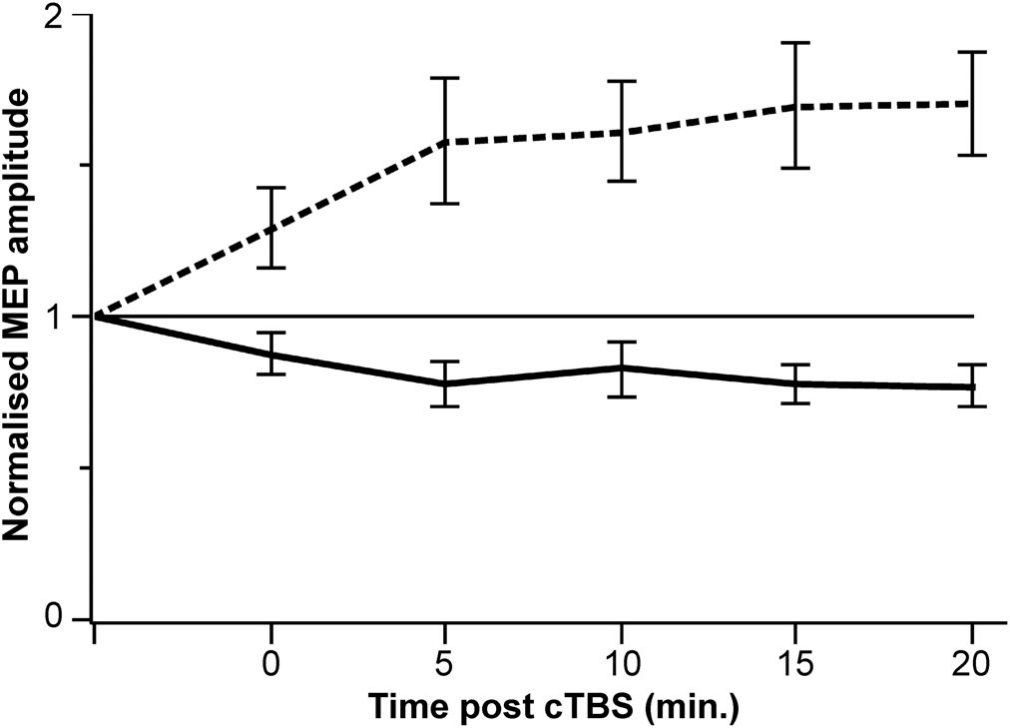
Time course of the normalized MEP amplitudes following cTBS at the motor cortex, plotted separately for the INH (solid line) and the NON‐INH (dotted line) subject groups. Error bars depict standard error of the mean. MEP, motor evoked potential

The magnitude of MEP before and after cTBS to the motor cortex gave us a qualitative measure of whether cTBS induced cortical inhibition (i.e., normalized grand average MEP amplitude of time interval 0–20 min after cTBS lower than MEP amplitude before cTBS), or not. In three subjects, we were unable to obtain an MEP measurement in some of the intervals between 0 and 20 min after cTBS. In two subjects, this was due to overheating of the TMS coil (3 out of 5 measurements missing in one subject, 1 out of 5 measurements missing in the other subject). In the remaining subject, this was due to the participant becoming nauseous and subsequently aborting the session (1 out of 5 measurements missing). In these subjects, we calculated the normalized grand average MEP amplitude using the available post‐cTBS intervals. In line with previous observations (Hamada et al., 2013; McAllister et al., 2013), our results indicated that 13 subjects showed evidence of cortical inhibition. The remaining 22 subjects did not show evidence of cortical inhibition (see Figure 4). Thus, 13 subjects were assigned to the inhibiting group (INH) and 22 subjects were assigned to the noninhibiting group (NON‐INH). A report of the side effects of cTBS that we observed can be found in the Supporting Information. The data underlying these results can be found at https://osf.io/gkysv/.

### 3.3 Effect of cTBS on performance in the TVA experiment

We used the horizontal EOG signal to assess whether saccades were present during stimulus presentation. On average, 99.5% (+/− 0.5%) of the trials were free from saccades. Trials with saccades were excluded from analysis. Following generally accepted guidelines, the quality of the TVA model fit, that is, how well the observed data fitted the predicted model, was assessed by the coefficient of determination *r* ^2^. If a fit revealed an *r* ^2^ below 60%, the data was separately inspected. For the whole report task, *r* ^2^ was above 90% for all subjects. For the partial report task, however, *r* ^2^ was below 60% for some subjects. In these subjects, the decision was made (through consensus between both authors) to exclude them from further analysis when (a) performance accuracy was below 40% in multiple conditions (indicating possible floor performance), and/or (b) visual inspection revealed a high deviation between observed and predicted values in multiple conditions (indicating a poor model fit). Importantly, this decision was made prior to the statistical analysis of the data, and the authors were blind with respect to the individual TVA parameter estimates and the group‐status (INH or NON‐INH) of the subjects during the decision‐making process. Of the four subjects excluded, three subjects demonstrated an *r* ^2^ of less than 30%, suggesting that the TVA parameter estimates would have been unlikely to be meaningful in these subjects.

Following cTBS to the vertex, the model fit yielded an average *r* ^2^ of 95% (*SD* = 2%) in the whole report task and 71% (*SD* = 12%) in the partial report task. Following cTBS at the IPS, the model fit yielded an average *r* ^2^ of 95% (*SD* = 2%) in the whole report task and 68% (*SD* = 15%) in the partial report task. Following cTBS at the TPJ, the model fit yielded an average *r* ^2^ of 95% (*SD* = 2%) in the whole report task and 71% (*SD* = 16%) in the partial report task. These model fits are comparable with those obtained in previous TVA‐based studies with young healthy participants (Finke et al., 2005; Matthias et al., 2009) and indicate a close correspondence between observed data and predicted model. The data underlying the results presented here and in the following sections can be found at https://osf.io/gkysv/.

#### 3.3.1 Effect of cTBS at the IPS

**Figure 5:**
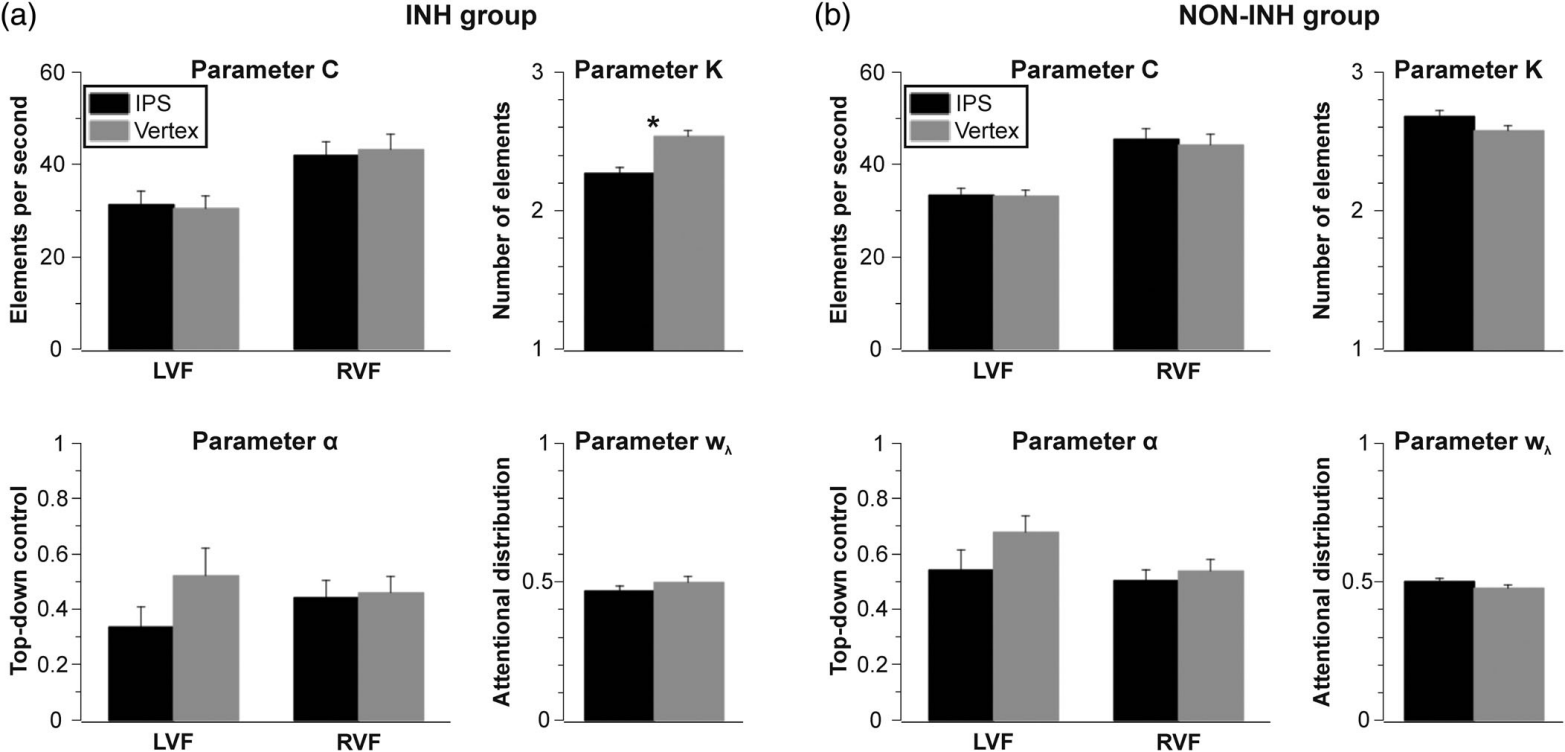
TVA parameter estimates following neuroinhibition at the IPS and vertex, plotted separately for the INH (a) and NON‐INH (b) group. * denotes *p* < .05. Error bars depict standard error of the mean as calculated from within‐subject variability (Loftus & Masson, 1994). IPS, intraparietal sulcus; TVA, theory of visual attention

We compared TVA task performance following cTBS at the IPS and TVA task performance following cTBS at the vertex to investigate which attentional sub‐processes were modulated after a transient inhibition of neural activity at the IPS. To statistically assess whether cTBS at the IPS modulated any of the TVA parameter estimates when compared to cTBS at the vertex, we performed mixed‐design ANOVAs with the within‐subject factor cTBS location (IPS or vertex) and the between‐subject factor group (INH or NON‐INH) for the TVA parameter estimates K and *w*_*λ*_, where we obtained a single parameter estimate for the entire visual field. For the TVA parameter estimates *C* and *α*, where we obtained separate parameter estimates for the left and right visual field, we performed mixed‐design ANOVAs with the within‐subject factors cTBS location (IPS or vertex) and stimulus location (left or right), and the between‐subject factor group (INH or NON‐INH). Figure 5 depicts the TVA parameter estimates following neuroinhibition at the IPS and vertex, separately for the INH (Figure 5a) and NON‐INH (Figure 5b) group.

For the TVA parameter estimate K, we found a significant interaction between the factors cTBS location and group (*F*_1,33_ = 11.279, *p* = .002, *p*_corr_ = .008, *ηp* ^2^ = .255). Neither the main effect of cTBS location (*F*_1,33_ = 2.111, *p* = .156, *p*_corr_ = .624, *ηp* ^2^ = .060) nor the main effect of group (*F*_1,33_ = 2.691, *p* = .110, *p*_corr_ = .440, *ηp* ^2^ = .075) was significant. To determine the source of this significant interaction, we performed separate paired‐samples *t*‐tests to assess the effect of cTBS location on TVA parameter estimate *K* for both the INH and the NON‐INH group. These analyses revealed that cTBS at the IPS significantly reduced the TVA parameter estimate *K* for the INH group (*t*_12_ = 3.442, *p* = .005, *p*_corr_ = .040, Cohen's *d* = 0.955), but not for the NON‐INH group (*t*_21_ = 1.472, *p* = .156, *p*_corr_ > .999, Cohen's *d* = 0.314). Thus, for the INH group only, neuroinhibition at the IPS induced a significant reduction of VSTM capacity.

We found no evidence to suggest that cTBS at the IPS significantly modulated any of the other TVA parameter estimates when compared to cTBS at the vertex. For the TVA parameter estimate *w*_*λ*_ neither the interaction between the factors cTBS location and group (*F*_1,33_ = 1.570, *p* = .219, *p*_corr_ = .876, *ηp* ^2^ = .045) nor any of the main effects (cTBS location: *F*_1,33_ = 0.026, *p* = .874, *p*_corr_ > .999, *ηp* ^2^ = .001; group: *F*_1,33_ = 0.046, *p* = .831, *p*_corr_ > .999, *ηp* ^2^ = .001) was significant. For the TVA parameter estimate *C*, we found a significant main effect of stimulus location (*F*_1,33_ = 66.498, *p* < .001, *p*_corr_ < .004, *ηp* ^2^ = .668), suggesting lower processing speed for targets in the left visual field than targets in the right visual field. However, neither the three‐way interaction of cTBS location by stimulus location by group (*F*_1,33_ = 0.466, *p* = .500, *p*_corr_ > .999, *ηp* ^2^ = .014) nor any of the two‐way interactions (cTBS location by group: *F*_1,33_ = 0.032, *p* = .859, *p*_corr_ > .999, *ηp* ^2^ = .001; stimulus location by group: *F*_1,33_ = 0.008, *p* = .931, *p*_corr_ > .999, *ηp* ^2^ < .001; cTBS location by stimulus location: *F*_1,33_ = 0.039, *p* = .845, *p*_corr_ > .999, *ηp* ^2^ = .001) nor any of the other main effects (cTBS location: *F*_1,33_ = 0.011, *p* = .918, *p*_corr_ > .999, *ηp* ^2^ < .001; group: *F*_1,33_ = 0.282, *p* = .599, *p*_corr_ > .999, *ηp* ^2^ = .008) was significant. For the TVA parameter α, neither the three‐way interaction of cTBS location by stimulus location by group (*F*_1,33_ = 0.119, *p* = .733, *p*_corr_ > .999, *ηp* ^2^ = .004) nor any of the two‐way interactions (cTBS location by group: *F*_1,33_ = 0.015, *p* = .903, *p*_corr_ > .999, *ηp* ^2^ < .001; stimulus location by group: *F*_1,33_ = 1.362, *p* = .252, *p*_corr_ > .999, *ηp* ^2^ = .040; cTBS location by stimulus location: *F*_1,33_ = 1.845, *p* = .184, *p*_corr_ = .736, *ηp* ^2^ = .053) nor any of the main effects (cTBS location: *F*_1,33_ = 2.504, *p* = .123, *p*_corr_ = .492, *ηp* ^2^ = .071; stimulus location: *F*_1,33_ = 0.500, *p* = .484, *p*_corr_ > .999, *ηp* ^2^ = .015; group: *F*_1,33_ = 4.102, *p* = .051, *p*_corr_ = .204, *ηp* ^2^ = .111) was significant.

Additionally, we compared the extinction index following cTBS at the IPS and cTBS at the vertex to investigate whether cTBS at the IPS modulated subjects' ability to attend to multiple simultaneously‐presented lateralized targets. As can be seen in Table 1, the extinction index was consistently around 0, suggesting absence of either contralateral or ipsilateral extinction‐like behavior in both the INH and the NON‐INH group following neuroinhibition at either the IPS or the vertex. To statistically assess whether cTBS at the IPS modulated multi‐target attention, we performed a mixed‐design ANOVA with the within‐subject factor cTBS location (IPS or vertex) and the between‐subject factor group (INH or NON‐INH). This analysis revealed neither a significant interaction between the factors cTBS location and group (*F*_1,33_ = 0.153, *p* = .698, *ηp* ^2^ = .005) nor a significant main effect of either cTBS location (*F*_1,33_ = 0.038, *p* = .847, *ηp* ^2^ = .001) or group (*F*_1,33_ = 0.774, *p* = .385, *ηp* ^2^ = .023), suggesting that cTBS at the IPS did not modulate multi‐target attention.

**Table 1:** Mean extinction index and associated standard error of the mean (in brackets and as calculated from within‐subject variability, see Loftus & Masson, 1994) following neuroinhibition at the IPS and vertex, separately for both the INH and the NON‐INH group

	IPS	Vertex
INH	−0.0088 (0.0303)	−0.0304 (0.0645)
NON‐INH	0.0184 (0.0219)	0.0257 (0.0219)

Some previous studies have, however, suggested that the effect of neuroinhibition on behavior can vary between individuals depending on baseline performance. For example, the results of Emrich, Johnson, Sutterer, and Postle (2017) suggest that the effect of neuroinhibition on attentional performance can vary as a function of individual differences in baseline VSTM capacity. To assess whether in the current study the effect of cTBS on the extinction index likewise depended on individual differences in baseline attentional performance, we performed exploratory analyses where we correlated the cTBS‐induced change in the extinction index (IPS minus vertex) with each of the baseline TVA parameter estimates obtained following cTBS at the vertex, in both the INH and the NON‐INH group. These Spearman's rank order correlation analyses, however, revealed no correlations that were significant at a Bonferroni‐corrected *p*‐value of .05/12 = .004 (see Table 2). This suggests that the absence of an effect of cTBS at the right IPS on extinction‐like behavior was not simply due to the effect being buffered by existing differences in baseline attentional performance.

**Table 2:** Results from the Spearman's rank order correlation analyses (correlations with uncorrected *p*‐value in brackets) to assess the correlation between the effect of cTBS at the IPS on the extinction index and each of the TVA parameter estimates in both the INH and the NON‐INH group

		*K*	*w*_*λ*_	*C* left	*C* right	*α* left	*α* right
INH (*n* = 13)	Effect of cTBS on extinction index	−.137 (.655)	.665 (.013)	−.489 (.090)	−.335 (.263)	−.099 (.748)	−.412 (.162)
NON‐INH (*n* = 22)	Effect of cTBS on extinction index	.310 (.160)	.306 (.166)	.072 (.751)	.201 (.371)	−.075 (.739)	−.114 (.615)

#### 3.3.2 Effect of cTBS at the TPJ

**Figure 6:**
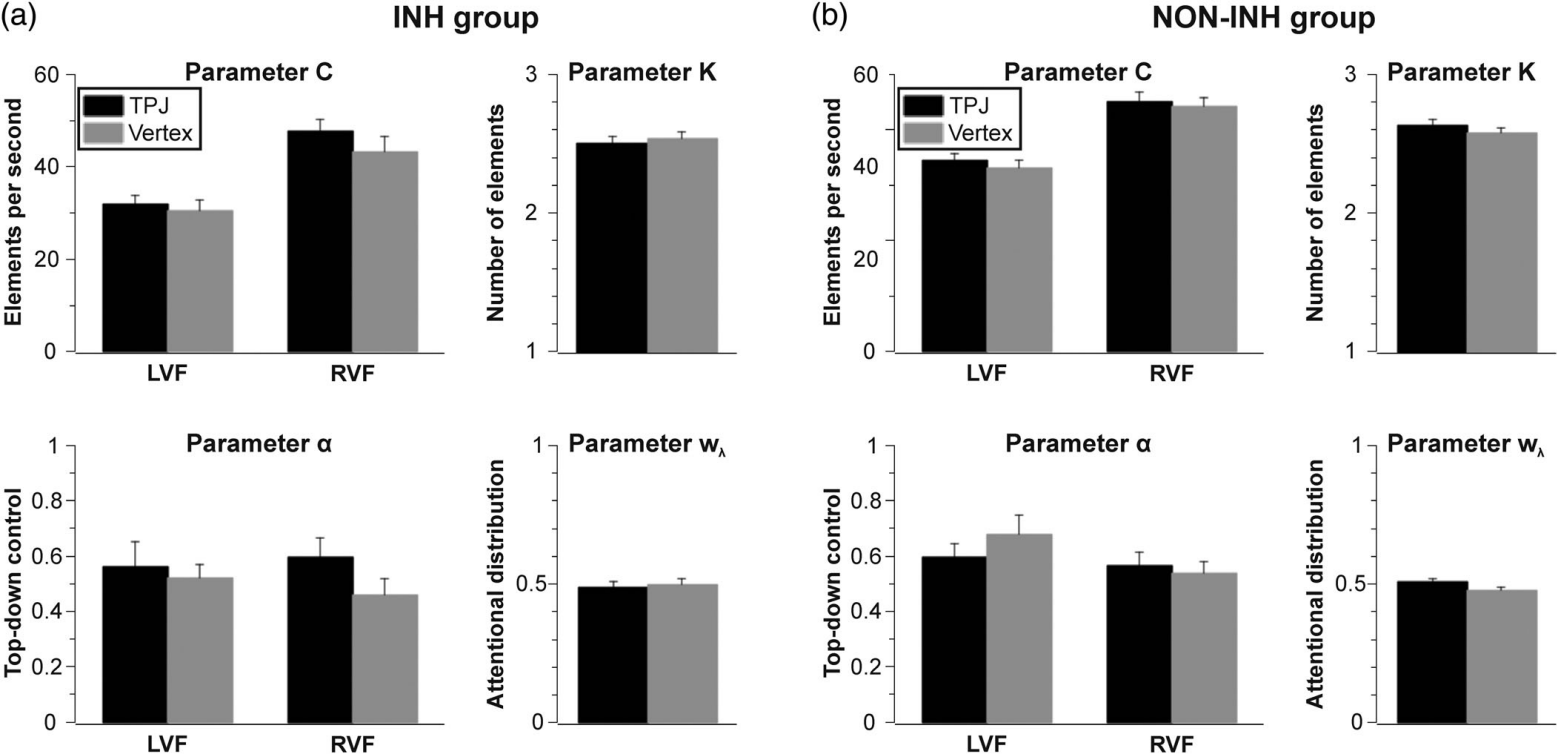
TVA parameter estimates following neuroinhibition at the TPJ and vertex, plotted separately for the INH (a) and NON‐INH (b) group. Error bars depict standard error of the mean as calculated from within‐subject variability (Loftus & Masson, 1994). TPJ, temporo‐parietal junction; TVA, theory of visual attention

Identically to the analyses performed to investigate the effects of cTBS at the IPS, we compared TVA task performance following cTBS at the TPJ and TVA task performance following cTBS at the vertex to investigate which attentional sub‐processes were modulated after a transient inhibition of neural activity at the TPJ. Figure 6 depicts the TVA parameter estimates following neuroinhibition at the TPJ and vertex, separately for the INH (Figure 6a) and NON‐INH (Figure 6b) group.

We found no evidence to suggest that cTBS at the TPJ significantly modulated any of the TVA parameter estimates when compared to cTBS at the vertex. For the TVA parameter estimates *K* and *w*_*λ*_ neither the interaction between the factors cTBS location and group (*K*: *F*_1,33_ = 0.530, *p* = .472, *p*_corr_ > .999, *ηp* ^2^ = .016; *w*_*λ*_: *F*_1,33_ = 0.670, *p* = .419, *p*_corr_ > .999, *ηp* ^2^ = .020) nor the main effect of cTBS location (*K*: *F*_1,33_ = 0.035, *p* = .852, *p*_corr_ > .999, *ηp* ^2^ = .001; *w*_*λ*_: *F*_1,33_ = 0.191, *p* = .665, *p*_corr_ > .999, *ηp* ^2^ = .006) nor the main effect of group (*K*: *F*_1,33_ = 0.448, *p* = .508, *p*_corr_ > .999, *ηp* ^2^ = .013; *w*_*λ*_: *F*_1,33_ = 0.001, *p* = .982, *p*_corr_ > .999, *ηp* ^2^ < .001) was significant. For the TVA parameter estimate C, we again found a significant main effect of stimulus location (*F*_1,33_ = 46.093, *p* < .001, *p*_corr_ < .004, *ηp* ^2^ = .583), suggesting lower processing speed for targets in the left visual field than targets in the right visual field. However, neither the three‐way interaction of cTBS location by stimulus location by group (*F*_1,33_ = 0.494, *p* = .487, *p*_corr_ > .999, *ηp* ^2^ = .015) nor any of the two‐way interactions (cTBS location by group: *F*_1,33_ = 0.315, *p* = .579, *p*_corr_ > .999, *ηp* ^2^ = .009; stimulus location by group: *F*_1,33_ = 0.832, *p* = .368, *p*_corr_ > .999, *ηp* ^2^ = .025; cTBS location by stimulus location: *F*_1,33_ = 0.347, *p* = .560, *p*_corr_ > .999, *ηp* ^2^ = .010) nor any of the other main effects (cTBS location: *F*_1,33_ = 1.419, *p* = .242, *p*_corr_ > .999, *ηp* ^2^ = .041; group: *F*_1,33_ = 0.024, *p* = .878, *p*_corr_ > .999, *ηp* ^2^ = .001) was significant. For the TVA parameter α, neither the three‐way interaction of cTBS location by stimulus location by group (*F*_1,33_ = 0.004, *p* = .949, *p*_corr_ > .999, *ηp* ^2^ < .001) nor any of the two‐way interactions (cTBS location by group: *F*_1,33_ = 0.817, *p* = .373, *p*_corr_ > .999, *ηp* ^2^ = .024; stimulus location by group: F_1,33_ = 0.721, *p* = .402, *p*_corr_ > .999, *ηp* ^2^ = .021; cTBS location by stimulus location: *F*_1,33_ = 1.079, *p* = .307, *p*_corr_ > .999, *ηp* ^2^ = .032) nor any of the main effects (cTBS location: *F*_1,33_ = 0.231, *p* = .634, *p*_corr_ > .999, *ηp* ^2^ = .007; stimulus location: *F*_1,33_ = 1.417, *p* = .242, *p*_corr_ = .968, *ηp* ^2^ = .041; group: *F*_1,33_ = 0.752, *p* = .392, *p*_corr_ > .999, *ηp* ^2^ = .022) was significant.

Additionally, identically to the analyses performed to investigate the effects of cTBS at the IPS, we compared the extinction index following cTBS at the TPJ and cTBS at the vertex to investigate whether cTBS at the TPJ modulated subjects' ability to attend to multiple simultaneously‐presented lateralized targets. As can be seen in Table 3, the extinction index was again consistently around 0, suggesting absence of extinction‐like behavior in both the INH and the NON‐INH group following neuroinhibition at either the TPJ or the vertex. Again, the mixed‐design ANOVA with the within‐subject factor cTBS location (TPJ or vertex) and the between subject factor group (INH or NON‐INH) revealed neither a significant interaction between the factors cTBS location and group (*F*_1,33_ = 0.896, *p* = .351, *ηp* ^2^ = .026) nor a significant main effect of either cTBS location (*F*_1,33_ = 0.062, *p* = .805, *ηp* ^2^ = .002) or group (*F*_1,33_ = 0.264, *p* = .611, *ηp* ^2^ = .008), suggesting that cTBS at the TPJ did not modulate multi‐target attention.

**Table 3:** Mean extinction index and standard error of the mean (in brackets and as calculated from within‐subject variability, see Loftus & Masson, 1994) following neuroinhibition at the TPJ and vertex, separately for both the INH and the NON‐INH group

	TPJ	Vertex
INH	−0.0042 (0.0389)	−0.0304 (0.0389)
NON‐INH	−0.0191 (0.0175)	0.0257 (0.0175)

**Figure 7:**
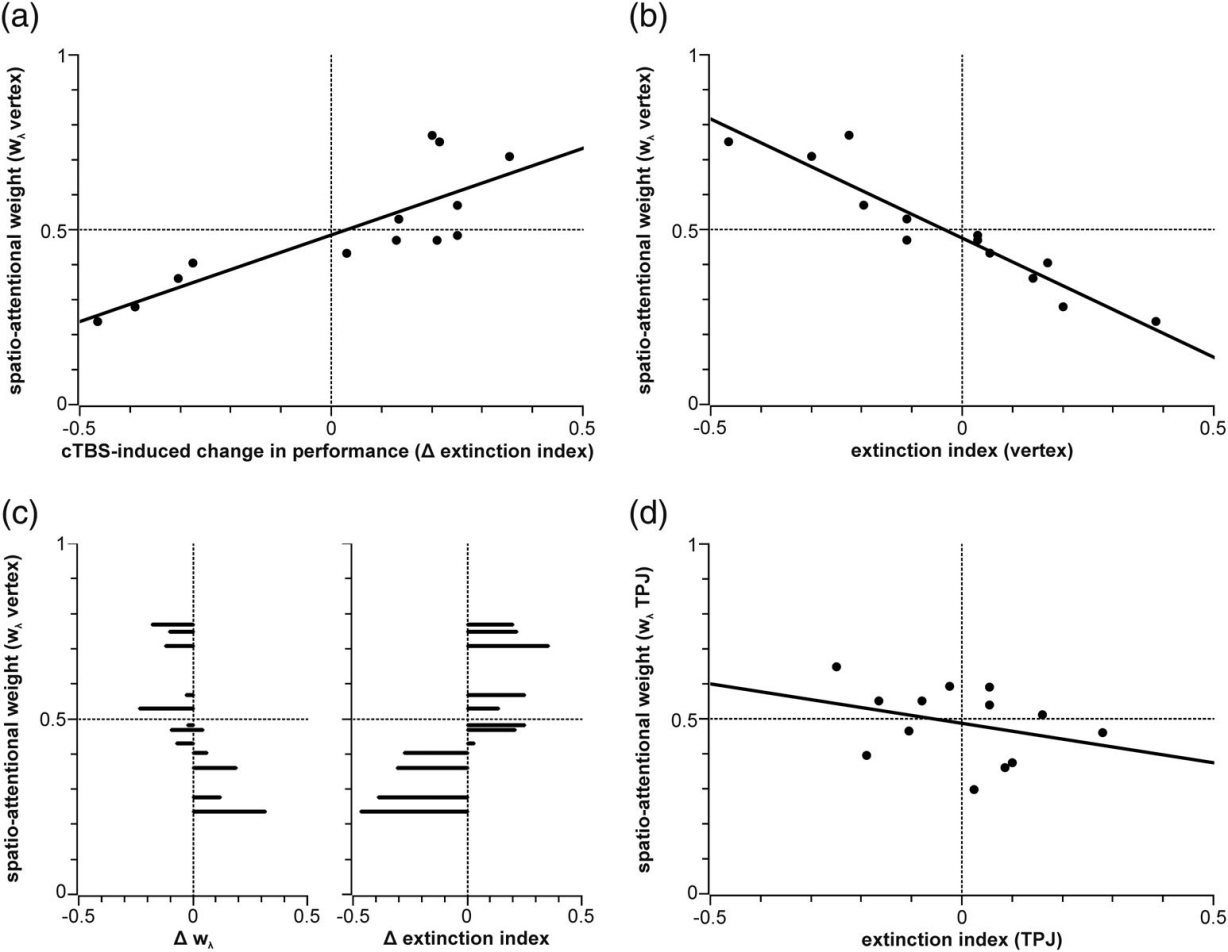
Effect of cTBS at the TPJ on multi‐target attention as a function of individual baseline spatio‐attentional weight (*w*_*λ*_) following cTBS at the vertex in the INH group. The cTBS‐induced change in the extinction index (TPJ minus vertex) correlated significantly with the baseline TVA parameter estimate *w*_*λ*_ following cTBS at the vertex (a). This effect, however, was mostly due to cTBS at the TPJ removing an effect that was present following cTBS at the vertex (b–d). Specifically, following cTBS at the vertex, there was a negative correlation between the extinction index and the TVA parameter estimate *w*_*λ*_ (b). Compared to this baseline, cTBS at the TPJ differentially modulated both the TVA parameter estimate *w*_*λ*_ and the extinction index as a function of the baseline TVA parameter estimate *w*_*λ*_ (c). As a consequence, the negative correlation between the extinction index and the TVA parameter estimate *w*_*λ*_ present following cTBS at the vertex was reduced following cTBS at the TPJ (d). TPJ, temporo‐parietal junction; TVA, theory of visual attention

To again assess whether the effect of cTBS on the extinction index depended on individual differences in baseline attentional performance, we performed exploratory analyses where we correlated the cTBS‐induced change in the extinction index (i.e., TPJ minus vertex) with each of the baseline TVA parameter estimates obtained following cTBS to the vertex, in both the INH and the NON‐INH group. These Spearman's rank order correlation analyses revealed a significant correlation between the effect of cTBS on the extinction index and the baseline TVA parameter estimate *w*_*λ*_ in the INH group (*r*_*s*_ = .847, *p* < .001, *p*_corr_ < .012, *r* ^2^ = .717; none of the other correlations were significant at a Bonferroni‐corrected *p*‐value of .05/12 = .004, see Table 4). Individuals with a baseline leftward spatio‐attentional bias (*w*_*λ*_ > .5) tended to show an increase in the extinction index following cTBS at the TPJ, whereas individuals with a baseline rightward spatio‐attentional bias (*w*_*λ*_ < .5) tended to show a decrease in the extinction index (see Figure 7a). A closer inspection of the data, however, revealed that this was mostly due to cTBS at the TPJ removing an effect that was present during the baseline. Specifically, following cTBS at the vertex (see Figure 7b), subjects with a leftward spatio‐attentional bias (*w*_*λ*_ > .5) showed a negative extinction index (i.e., extinction‐like behavior for targets in the right visual field), whereas subjects with a right‐ward spatio‐attentional bias (*w*_*λ*_ < .5) showed a positive extinction index (i.e., extinction‐like behavior for targets in the left visual field). Compared to this baseline, cTBS at the TPJ elicited a decrease in the TVA parameter estimate *w*_*λ*_ for subjects with a baseline left‐ward spatio‐attentional bias, which was accompanied by an increase in the extinction index (see Figure 7c). For subjects with a baseline right‐ward spatio‐attentional bias, on the other hand, cTBS at the TPJ elicited an increase in the TVA parameter estimate *w*_*λ*_, which was accompanied by a decrease in the extinction index. Regardless of the direction of the baseline spatio‐attentional bias, cTBS at the TPJ thus led to a shift of the TVA parameter estimate *w*_*λ*_ toward a value of 0.5 (reflecting equal attentional weighting of both visual fields), which was accompanied by a shift of the extinction index toward 0 (Figure 7d). In other words, for both the TVA parameter estimate *w*_*λ*_ and the extinction index, cTBS at the TPJ (compared to cTBS at the vertex) led to a reduction of behavioral variance without affecting the behavioral mean. Had the effect of cTBS on the extinction index depended on individual differences in baseline attentional performance, we would have expected cTBS at the TPJ to instead have increased behavioral variance. Thus, this again suggests that the absence of an effect of cTBS at the right TPJ on extinction‐like behavior was not simply due to the effect being buffered by existing differences in baseline attentional performance.

**Table 4:** Results from the Spearman's rank order correlation analyses (correlations with uncorrected *p*‐value in brackets) to assess the correlation between the effect of cTBS at the TPJ on the extinction index and each of the TVA parameter estimates in both the INH and the NON‐INH group

		*K*	*w*_*λ*_	*C* left	*C* right	*α* left	*α* right
INH (*n* = 13)	Effect of cTBS on extinction index	−.220 (.470)	.847 (<.001)	−.501 (.081)	−.338 (.258)	−.085 (.782)	−.715 (.006)
NON‐INH (*n* = 22)	Effect of cTBS on extinction index	.049 (.828)	.273 (.218)	−.139 (.537)	−.368 (.092)	.152 (.500)	.046 (.838)

## 4 DISCUSSION

In the current study, we aimed to elucidate the role of the IPS and the TPJ in our ability to attend to multiple simultaneously‐presented lateralized targets, and the failure of this ability in extinction patients. In neurologically healthy subjects, we combined TVA with fMRI‐guided cTBS to transiently inhibit neural activation at either the right IPS or the right TPJ. Specifically, we used fMRI to individually define the location of the right IPS and right TPJ area associated with multi‐target attention. Subsequently, we assessed the effect of cTBS‐induced transient neuroinhibition at these brain areas on attentional sub‐processes as modeled using TVA, as well as multi‐target attention. Our study yielded several important findings.

### 4.1 Effect of cTBS at the right IPS

Our results suggest that, in subjects responsive to cTBS, a transient inhibition of neural activity at an area of the right IPS associated with multi‐target attention elicits a reduction of VSTM capacity. Previous studies have suggested a role for the IPS in multi‐target attention (Çiçek et al., 2007; de Haan, Bither, et al., 2015; Geng et al., 2006) and its failure in extinction patients (Battelli et al., 2009; Cazzoli et al., 2009; Hilgetag et al., 2001; Pascual‐Leone et al., 1994), as well as VSTM capacity (Jeong & Xu, 2013; Todd & Marois, 2004; Xu & Chun, 2006). The combination of fMRI‐guided cTBS and TVA in our study allowed us to extend these previous observations. Specifically, our results suggest that, on a within‐subject level, multi‐target attention and VSTM capacity are intimately and causally linked in the right IPS. This is in line with previous demonstrations of a near‐perfect behavioral correlation (*r* values > .8) between the amount of targets subjects can attend and the amount of target representations subjects can encode and maintain in VSTM (Tsubomi, Fukuda, Watanabe, & Vogel, 2013). Moreover, this is also in line with previous demonstrations that, on a within‐subject level, neural activity in the same part of the IPS simultaneously correlates both with the ability to attend to multiple simultaneously‐presented targets and with the ability to encode and maintain multiple target representations in VSTM (Emrich et al., 2011; Mitchell & Cusack, 2008).

More generally, our results suggest that the right IPS is critically associated with a general capacity‐limited encoding mechanism, that is, engaged regardless of whether targets have to be attended or remembered. In our study, both the partial and whole report tasks, as well as the fMRI object identification task, used immediate report. Thus, these tasks required target encoding, but only minimal target maintenance and/or storage. As such, our finding that a transient inhibition of neural activity at an area of the right IPS localized with the fMRI object identification task elicits a reduction in VSTM capacity as measured with the whole report task suggests that, in our study, cTBS at the right IPS impaired target encoding mechanisms that underlie both multi‐target attention and VSTM capacity. This is in line with previous observations that suggest that VSTM capacity limitations in the IPS can be traced back to encoding limitations (Buschman, Siegel, Roy, & Miller, 2011), and that the relationship between VSTM capacity and neural activity in the IPS breaks down with longer delay periods that presumably cause performance to rely more heavily on target maintenance and storing mechanisms (Magen, Emmanouil, McMains, Kastner, & Treisman, 2009). Moreover, this view that the IPS is associated with a general capacity‐limited encoding mechanism is also supported by observations of similar capacity limitations in the IPS during enumeration (Knops, Piazza, Sengupta, Eger, & Melcher, 2014) and multiple object tracking (Howe, Horowitz, Morocz, Wolfe, & Livingstone, 2009).

Curiously, a transient inhibition of neural activity at an area of the right IPS associated with multi‐target attention did not elicit extinction‐like behavior in our study. This contrasts with the results from several previous studies that suggest that transient neuroinhibition at the IPS is capable of eliciting extinction‐like behavior (Battelli et al., 2009; Cazzoli et al., 2009; Hilgetag et al., 2001; Pascual‐Leone et al., 1994). As mean performance accuracy was below 80% in all conditions of the partial report task (regardless of cTBS location), our failure to find evidence of extinction‐like behavior cannot simply be attributed to ceiling effects. Moreover, the results of our exploratory correlation analyses suggest that the effect of cTBS at the right IPS on extinction‐like behavior was not simply buffered by existing differences in baseline attentional performance. A possible explanation for our failure to elicit extinction‐like behavior is that a nonlateralized attentional deficit that is not accompanied by a lateralized attentional deficit is not sufficient to elicit extinction‐like behavior. Extinction is currently seen as a consequence of both lateralized and nonlateralized attentional deficits (de Haan et al., 2012; Driver et al., 1997; Karnath, 1988; see also for example, Danckert & Ferber, 2006 who propose the same for neglect). In other words, for extinction to occur, both lateralized and nonlateralized attentional deficits may need to be present. In our study, cTBS at the right IPS elicited a nonlateralized reduction in VSTM capacity. We did not, however, find any evidence to suggest that cTBS at the right IPS elicited a lateralized attentional deficit. Together with the results of a previous study, where we found that the presence of a lateralized attentional deficit alone is also not sufficient to elicit extinction (de Haan, Stoll, et al., 2015), this supports the idea that neither lateralized, nor nonlateralized attentional deficits are sufficient to elicit extinction‐like behavior on their own. The previous TMS studies that did find extinction‐like behavior following transient neuroinhibition at the IPS (Battelli et al., 2009; Cazzoli et al., 2009; Hilgetag et al., 2001; Pascual‐Leone et al., 1994), typically targeted locations slightly more posterior to the location that we targeted. These more posterior areas of the IPS have been suggested to be involved not only with VSTM capacity, but also with spatial attention (Sheremata, Bettencourt, & Somers, 2010; Sheremata & Silver, 2015; Sheremata, Somers, & Shomstein, 2018). Thus, by targeting these more posterior areas of the IPS, it is possible that these previous TMS studies were capable of eliciting extinction‐like behavior by bringing about both a nonlateralized reduction in VSTM capacity, as well as a lateralized spatio‐attentional bias. A closer look at other previous studies does indeed suggest that the areas of the brain where fMRI activity is higher during situations where subjects attend and respond to multiple simultaneously‐presented lateralized targets than during situations where subjects attend and respond to single lateralized targets are located more anteriorly in the IPS than the areas of the brain where transient neurodisruption leads to impaired multi‐target attention (see for example, fig. 3 in de Haan et al., 2012). Overall, this supports previous suggestions that different areas of the right IPS may provide different contributions to our ability to attend to multiple simultaneously‐presented lateralized targets (de Haan, Bither, et al., 2015).

We were not able to replicate the results from Hung et al. (2005) and Moos et al. (2012) that suggest that the right IPS contributes to top‐down attentional control. In the current study, a cTBS‐induced transient inhibition of neural activity at the right IPS failed to elicit a significant modulation of this top‐down control parameter. Nevertheless, in line with the results from Moos et al. (2012), we did observe a numerical trend toward an increase of top‐down control in the contralateral visual field (numerically lower parameter *α* values) following transient neuroinhibition at the right IPS, particularly in subjects responsive to cTBS. That is, the negative effect of adding a distractor letter to the display on target letter performance was numerically reduced following cTBS at the IPS. Such counterintuitive facilitatory effects of neuroinhibition have previously been interpreted as a consequence of a decrease in the global cortical excitation level (Antal et al., 2004; Moos et al., 2012). In the absence of statistically significant effects, however, our results here should be interpreted with caution.

### 4.2 Effect of cTBS at the right TPJ

We failed to find evidence that a transient inhibition of neural activity at the right TPJ modulates any of the TVA parameters or extinction‐like behavior. As mean performance accuracy was below 80% in all conditions of the partial report task and the mean number of letters correctly reported was below 3 (out of 5) even at the longest stimulus exposure duration used in the whole report task (regardless of cTBS location), our failure to find evidence of impaired attentional selection or extinction‐like behavior cannot simply be attributed to ceiling effects. Moreover, the results of our exploratory correlation analyses again suggest that the effect of cTBS at the right TPJ on extinction‐like behavior was not simply buffered by existing differences in baseline attentional performance. Our failure to find evidence of impaired attentional selection or extinction‐like behavior following a transient inhibition of neural activity at the right TPJ is in direct contrast with the results from a multitude of studies that argue for an important role of the TPJ in attentional selection (Duncan et al., 1999; Habekost & Rostrup, 2006; Peers et al., 2005) and our ability to attend to multiple simultaneously‐presented lateralized targets (Chechlacz et al., 2013; Grandjean et al., 2008; Karnath et al., 2003; Meister et al., 2006; Ticini et al., 2010).

One possible explanation for our null results is that, in contrast to the suggestions from previous studies, the right TPJ does not play a role in attentional selection and multi‐target attention. This would explain the repeated observation (Çiçek et al., 2007; de Haan, Bither, et al., 2015; Geng et al., 2006) that comparing the fMRI activation during situations where subjects attend and respond to multiple simultaneously‐presented lateralized targets to the fMRI activation during situations where subjects attend and respond to single lateralized targets readily isolates the IPS, but not the TPJ (but see Beume et al., 2015 for an exception). The results from patient and transient neuroinhibition studies that implicate the TPJ in attentional selection and multi‐target attention could then be explained by postulating that the behavioral effects seen after a (transient) inhibition of neural activity at the TPJ, are actually a consequence of remote dysfunction (e.g., diaschisis‐like effects, Feeney & Baron, 1986; see also Umarova et al., 2011). A direct test of this idea could be performed using for example concurrent TMS‐fMRI to investigate the distal functional effects of transient neuroinhibition at the TPJ and their relation to attentional selection and multi‐target attention. To the best of our knowledge, however, such a study has not been conducted so far.

Another possible explanation for our null results is that the right TPJ does play a role in attentional selection and multi‐target attention, but that our study simply failed to detect this. One potential cause of our failure to detect the involvement of the right TPJ in attentional selection and multi‐target attention, is that our cTBS protocol failed to induce the desired neural effect (de Graaf & Sack, 2011). To minimize this possibility, however, we obtained a qualitative measurement of the effect of cTBS on cortical functioning at the motor cortex in each individual subject prior to the TVA sessions. Importantly, our results suggest that that these neural effect of cTBS at the right motor cortex generalized to the right IPS, as only those subjects that showed evidence of neural inhibition following cTBS to the motor cortex, showed reduced task performance (and thus evidence of neural inhibition) following cTBS at the IPS (see also Section 4.3). Nevertheless, we cannot exclude the possibility that our cTBS protocol was less effective at the right TPJ than at the right IPS.

Another potential cause of our failure to detect the involvement of the right TPJ in attentional selection and multi‐target attention is that we applied cTBS at the wrong part of the TPJ. The TPJ lacks a clear functional or anatomical definition and likely consists of multiple subunits with separate functions (Bzdok et al., 2013; Krall et al., 2015; Mars et al., 2012). We might thus simply have failed to inhibit the attentionally‐relevant subarea. Our aim was to apply cTBS at a subarea of the right TPJ associated with multi‐target attention. As such, the intuitive choice would have been to functionally localize the right TPJ by comparing the fMRI activation during situations where subjects attend and respond to multiple targets to the fMRI activation during situations where subjects attend and respond to single targets. As stated above, however, this comparison has generally shown to be incapable of functionally localizing the TPJ (Çiçek et al., 2007; de Haan, Bither, et al., 2015; Geng et al., 2006). A few studies have, however, demonstrated that a reduction of VSTM capacity by a concurrent VSTM maintenance task is capable of eliciting a functional deactivation of the TPJ (Emrich et al., 2011; Todd et al., 2005), which has in turn been associated with impaired target detection in both single‐target (Todd et al., 2005) and multi‐target environments (Emrich et al., 2011). On the basis of these studies, we hypothesized that it might be feasible to functionally localize the subarea of the TPJ associated with multi‐target attention by comparing the fMRI activation during a high‐load VSTM maintenance task to the fMRI activation during a low‐load VSTM maintenance task. In our group analysis, the MNI coordinates of our TPJ cluster, as defined by the subtraction of the high‐load conditions from the low‐load conditions, were 50–5428. Meister et al. (2006) applied TMS at MNI coordinates close to our TPJ coordinates and did find extinction‐like behavior. Nevertheless, given the ambiguity concerning the precise anatomical location of the TPJ in general, and attentionally‐relevant subareas of the TPJ in particular, we cannot exclude the possibility that we failed to inhibit the “correct” part of the right TPJ.

### 4.3 Effect of cTBS on cortical function

Finally, our results suggest that individually determining the neural effect of cTBS at the motor cortex may provide meaningful information concerning the presence or absence of neuroinhibition following cTBS in cortical areas beyond the motor cortex. Recent studies suggest that only 42% (Hamada et al., 2013) to 50% (McAllister et al., 2013) of subjects show the expected neural response of inhibition of cortical function following cTBS at the motor cortex. This implies that, when using cTBS to inhibit cortical function in areas beyond the motor cortex, the expected neural effect might not be present in all subjects. If true, this not only complicates the subsequent interpretation of research findings, but also severely reduces the statistical power to detect an effect in these studies. An intuitive solution would be to measure the neural effect of cTBS at the region(s) of interest in each individual subject prior to the main study and group subjects according to whether they show the desired neural effect or not. Unfortunately, whereas at the motor cortex the neural effect of cTBS can be assessed directly, for example by obtaining MEPs, this is not possible for many of the cortical areas that are of interest to scientists aiming to investigate higher cognitive functions such as selective attention.

In the current study, we hypothesized that the neural effect of cTBS at the motor cortex might generalize to our areas of interest (IPS and TPJ) beyond the motor cortex. Thus, prior to our main TVA sessions, we measured the effect of cTBS at the motor cortex and, in each individual subject, assessed whether cTBS resulted in an inhibition of cortical function. Subsequently, we separately assessed the behavioral effects of cTBS at our regions of interest for the subjects that showed evidence of neural inhibition following cTBS at the motor cortex and those that did not. In our study, 37% (13 out of 35 subjects) of the subjects demonstrated neural inhibition following cTBS at the motor cortex, which is in line with what Hamada et al. (2013) and McAllister et al. (2013) reported following the same stimulation protocol. Interestingly, our results suggest that the neural effect of cTBS at the motor cortex might generalize to cortical areas beyond the motor cortex. Specifically, only those subjects who displayed the expected inhibitory cortical response following cTBS at the motor cortex, showed evidence of reduced behavioral performance following cTBS at the IPS. This suggests that the neural effect of cTBS measured at the motor cortex may also provide meaningful information concerning the neural effect of cTBS at areas beyond the motor cortex. As such, these results suggest that future cTBS studies aiming to investigate the effect(s) of cTBS at cortical areas beyond the motor cortex might benefit from assessing the individual neural effect of cTBS at the motor cortex in a separate session before the main experiment.

## Supporting information


**Appendix S1:** Supplementary Information

## Data Availability

The data underlying the results can be found at https://osf.io/gkysv/ (https://doi.org/10.17633/rd.brunel.7378418.v1).
