# Supplementary material for: Multi‐target attention and visual short‐term memory capacity are closely linked in the intraparietal sulcus
Source: Hum Brain Mapp. 2019 May 6;40(12):3589–605. doi: 10.1002/hbm.24618 (PMC6767774; doi:10.1002/hbm.24618)
Supplement: Supplementary file 1 — Appendix S1: Supplementary Information [file HBM-40-3589-s001.docx]

## Supplementary Methods

## *fMRI localiser*

Subjects performed a combined visual short-term memory (VSTM) and object identification task, based on the task used by Emrich et al. (2011), while lying in the bore of the MRI scanner. The software package E-Prime (Psychology Software Tools Inc., Sharpsburg, PA, USA) running on a PC was used to present the stimuli. Subjects were first presented with a fixation cross of 0.7° visual angle for a variable duration (350–7850 ms). This was followed by a VSTM sample display consisting of either 1 (low VSTM load) or 3 (high VSTM load) coloured discs that could appear at 8 possible locations surrounding the central fixation cross for a duration of 150 ms. Discs subtended 0.38° visual angle and were separeated by a minimum of 0.24° visual angle. Subjects were instructed to maintain both location and colour of the disc(s) presented in VSTM. Following this VSTM sample display, subjects were presented with an object sample display for a duration of 67 ms, containing either a single object in the left or the right visual field or 2 objects, one in each visual field. Objects subtended 1°x1° visual angle and were presented at an eccentricity of 10° visual angle at the horizontal midline. This object sample display was immediately followed by an object probe display, presented for a duration of 3000 ms, that contained 1 object in each visual field. Subjects were instructed to indicate which object(s) from the object probe display matched the object(s) shown in the object sample display (left, right, both, or none) by pressing the appropriate button on a button box held in their right hand. Finally, following the object probe display, subjects were presented with a VSTM probe display for a duration of 2000 ms, that contained 1 coloured disc at one of the eight possible locations surrounding the central fixation cross. Subjects were instructed to indicate whether or not the colour and position of the coloured disc in the VSTM probe display matched the colour and position of (one of) the coloured disc(s) presented in the VSTM sample display displayed at the start of the trial, again by pressing the appropriate button on a button box held in their right hand. Thus, the object identification task was performed either under conditions of low VSTM load, or under conditions of high VSTM load. As such, the resulting object presentation conditions were: bilateral-object_low-load, left-object_low-load, right-object_low-load, bilateral-object_high-load, left-object_high-load, and right-object_high-load.

In total, subjects performed 288 trials, divided over 6 runs (48 trials per run) while in the MRI scanner. As a consequence, each condition was presented 8 times per run. Throughout the entire task, subjects were instructed to lie as still as possible and to fixate the central fixation cross. Moreover, subjects were instructed to prioritise the VSTM task over the object identification task. In addition to the 6 runs of the task performed in the scanner, subject performed 1 run of this task outside of the scanner to familiarise themselves with the task. In the MRI scanner, eye fixation position was continuously measured at 50 Hz with an MR-compatible eye-tracker (SensoMotoric Instruments) and a head restraint was be used to minimize head movement. The eye tracking signal was analyzed using the program iLab (Gitelman 2002) and Matlab 2014a (Mathworks, Inc., Natick, MA, USA). On a trial by trial basis we calculated the duration that subjects spent ﬁxated within 1.5° visual angle radius of the center of either the ﬁxation cross or the peripheral target boxes as a percentage of the total experimental duration after removing eye blinks. A failure to ﬁxate was deﬁned as spending <90% of the total experimental duration ﬁxated on the ﬁxation cross and/or spending >1% of the total experimental duration ﬁxated on either the left or the right peripheral target box.

While subjects performed the combined VSTM and object identification task, the fMRI signal was continuously measured. All functional imaging was performed using a 3T Siemens Magnetom Trio scanner (Erlangen, Germany). For each subject, 6 sessions of continuous fMRI data were collected (one for each task run). Each session consisted of a series of whole-brain functional T_2_* EPI volumes covering the entire task run duration. The fMRI volumes were collected axially with a flip angle of 90°, a time to echo (TE) of 40 ms and a time to repetition (TR) of 2680 ms. Each fMRI volume contained 33 slices acquired in sequential ascending order with 3 mm^3^ voxel size without gap between slices (field of view [FOV]: 192x192). Additionally, for each subject we sagitally acquired a T_1_-weighted Magnetization Prepared Rapid Gradient Echo (MP-RAGE) anatomical volume (176 slices, 1x1x1 mm, 240x256 FOV) with a flip-angle of 15°, a TE of 3.4 ms and a TR of 2000 ms to aid normalisation and visualisation of the functional data.
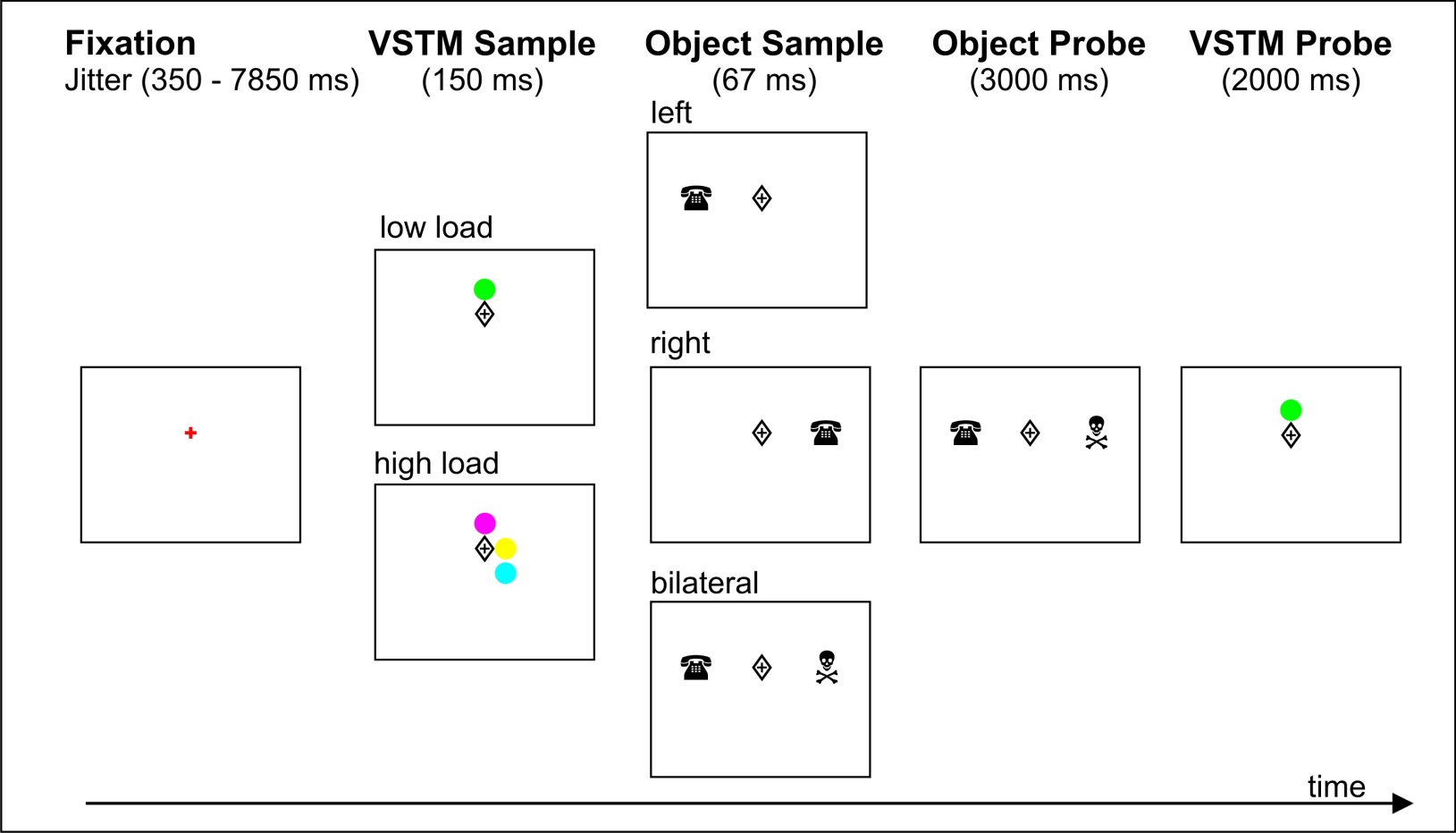

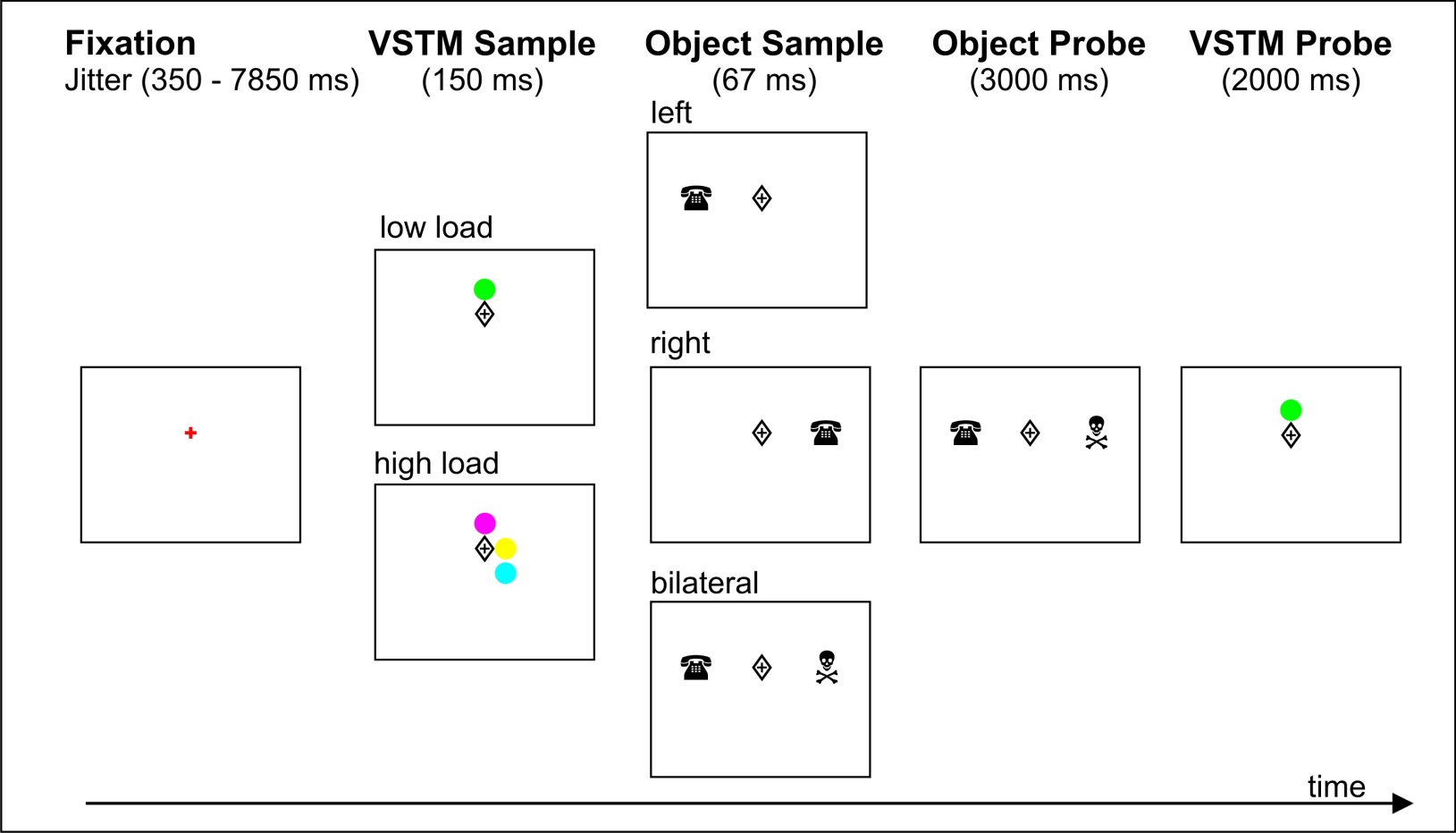
Preprocessing and statistical analyses were performed with SPM8 (Wellcome Department of Imaging Neuroscience, London, UK) implemented in Matlab 2014a (Mathworks, Inc., Natick, MA, USA). The functional volumes were slice time corrected using the middle slice as the reference slice (Henson et al. 1999) and realigned to match the first volume of the session (Friston et al. 1995). Two runs with scan to scan movements larger than 1 voxel size (3 mm) were excluded from analyses. Subsequently, the T1-weighted volume was coregistered with the mean functional volume obtained after realignment (Collignon et al. 1995). Transforms for warping the coregistered T1-weighted volume to standard stereotaxic space were computed using the unified segmentation & normalisation approach (Ashburner and Friston 2005). The resulting transformation parameters were used to warp the functional volumes and structural volumes into stereotaxic space. Finally, the functional volumes were spatially smoothed with an isotropic 8 mm full-width at half-maximum Gaussian filter. Each object presentation condition (bilateral_low-load, left_low-load, right_low-load, bilateral_high-load, left_high-load, right_high-load) was modelled using the standard SPM8 haemodynamic response function (Friston et al. 1995; Lange 2000; Worsley 2001; Kiebel and Holmes 2003) with temporal and dispersion derivatives. To reduce global noise, the time series of the mean white matter signal was added as a regressor. The inclusion of this regressor has recently been shown to improve the sensitivity of statistical analyses without inducing artificial signal changes by itself (Linzenbold and Himmelbach 2012).

In each individual subject, we defined the location of the right IPS area associated with multi-target attention by performing a conjunction analysis comparing the neural activity elicited by bilateral object sample displays to the neural activation elicited by unilateral object sample displays ([(bilateral > unilateral left) AND (bilateral > unilateral right)], analogously to the approach taken in de Haan et al (2015), averaged over both VSTM loads. The location of the right TPJ area associated with multi-target attention was defined by subtracting the neural activation elicited by object sample displays presented during low VSTM load from the neural activity elicited by object sample displays presented during high VSTM load, averaged over both unilateral and bilateral object presentation conditions. For both the IPS and the TPJ, the relevant contrast was thresholded at a voxel-based threshold of p < .05 (Bonferroni FWE corrected for multiple comparisons), and the coordinates of the highest peak associated with the relevant contrast within either the right posterior parietal cortex (for the IPS) or the inferior parietal lobe / superior temporal gyrus (TPJ) were extracted. Finally, the coordinates of the functionally defined IPS and TPJ were transformed from stereotaxic MNI space to native space using the deformation field image created in the normalization of the T1-weighted volume.

**Supplementary Results**

*Behavioural results from the fMRI localiser*

VSTM task. We calculated VSTM capacity using the formula developed by Pashler (1988) and modified by Cowan (2001) that combines accuracy and VSTM load: K = load * (hits + correct rejections -1). Subjects remembered significantly more coloured discs in the high load (1.7) than in the low load (0.9) condition (t (34) = 8.94, p < 0.0001). As in the study by Emrich et al. (2011), the relatively low performance in the high load condition is most likely a result of the dual-task environment.

Object identification task. Performance accuracies for each of the object identification conditions can be found in Supplementary Table I. To statistically assess performance accuracies in the object identification task, we conducted a 2 (load: high, low) x 2 (object presentation: unilateral, bilateral) x 2 (target location: left, right) repeated measures ANOVA. Object identification accuracy was significantly lower during high than during low VSTM load conditions (F (1,34) = 18.2, p = 0.0002), suggesting that subjects prioritised the VSTM task as instructed. Additionally, object identification accuracy was significantly lower during bilateral than during unilateral object presentation conditions (F(1,34) = 39.1, p < 0.0001). Neither the main effect of target location, nor any of the interaction effects reached significance.

Supplementary Table I: Mean accuracy (%) and normalised standard error (in brackets) for each of the object identification conditions in the VSTM localiser (Loftus & Masson, 1994).

| **low load** | | | | **high load** | | | |
| --- | --- | --- | --- | --- | --- | --- | --- |
| **unilateral** | | **bilateral** | | **unilateral** | | **bilateral** | |
| **left** | **right** | **left** | **right** | **left** | **right** | **left** | **right** |
| 95 (1) | 96 (1) | 89 (1) | 91 (1) | 91 (1) | 91 (1) | 87 (1) | 87 (1) |

Eye-tracking data. Due to technical problems the eye tracking signal of two subjects could not be recorded. Additionally, in eight subjects the eye tracking signal could not be analysed offline due to bad data quality. In these cases, we conducted online fixation control. Here, in all subjects fixation was stable throughout the experiment. Offline analysis of the eye tracking signal in the remaining 25 out of 35 subjects demonstrated that subjects spend 98.96% (standard deviation, SD 0.91%) of the stimulus presentation time fixating the central fixation cross. The left target position was fixated for 0.01% (SD 0.03%) and the right target for 0.02% (SD 0.02%) of the stimulus presentation time.

## *Side effects of cTBS*

Most of our subjects reported no side effects after TMS (single-pulse TMS and cTBS). Eight of the 35 subjects reported one or two of the following symptoms: headache, tiredness, nausea and dizziness. In 4 subjects the reason was probably a too tight headband, which was necessary for neuronavigation. After removing the headband, the symptoms disappeared immediately. One subject reported nausea during the first session, the other sessions were without side effects. The remaining 3 subjects reported symptoms of headache, nausea, dizziness or tiredness in the afternoon after the TMS stimulation in 1 or 2 of the 4 TMS sessions. During the stimulation muscle twitches were common but bearable and the stimulation intensity was on average 41% (SD 6%) of the maximum stimulator output.

**References**

Ashburner J, Friston KJ. 2005. Unified segmentation. NeuroImage. 26:839–851.

Collignon A, Maes F, Delaere D, Vandermeulen D, Suetens P, Marchal G. 1995. Automated multi-modality image registration based on information theory. In: Bizais Y, Barillot C, Di Paola R, editors. Information processing in medical imaging. Dordrecht, The Netherlands: Kluwer Academic Publishers. p. 263-274.

Cowan N. 2001. The magical number 4 in short-term memory: a reconsideration of mental storage capacity. Behav Brain Sci. 24:87-114; discussion 114-185.

Emrich SM, Burianova H, Ferber S. 2011. Transient perceptual neglect: Visual working memory load affects conscious object processing. J Cogn Neurosci. 23:2968–2982.

Friston KJ, Ashburner J, Frith CD, Poline JB, Heather JD, Frackowiak RSJ. 1995. Spatial registration and normalization of images. Hum Brain Mapp. 3:165–189.

Friston KJ, Holmes AP, Worsley KJ, Poline J-P, Frith CD, Frackowiak RSJ. 1995. Statistical parametric maps in functional imaging: A general linear approach. Hum Brain Mapp. 2:189–210.

Gitelman DR. 2002. ILAB: A program for postexperimental eye movement analysis. Behav Res Methods Instrum Comput. 34:605–612.

de Haan B, Bither M, Brauer A, Karnath H-O. 2015. Neural correlates of spatial attention and target detection in a multi-target environment. Cereb Cortex 25:2321–2331.

Henson R, Buechel C, Josephs O, Friston K. 1999. The slice-timing problem in event-related fMRI. NeuroImage 9:125–125.

Kiebel SJ, Holmes AP. 2003. The general linear model. In: Frackowiak RSJ, Friston KJ, Frith C, Dolan R, Price CJ, Zeki S, Ashburner J, Penny WD, editors. Human brain function. 2nd ed. San Diego: Academic Press. p. 725-760.

Lange N. 2000. Statistical procedures for functional MRI. In: Moonen CTW, Bandettini PA, editors. Functional MRI. Heidelberg: Springer-Verlag Berlin. p. 301–335.

Linzenbold W, Himmelbach M. 2012. Signals from the deep: Reach-related activity in the human superior colliculus. J Neurosci. 32:13881–13888.

Loftus GR, Masson ME. 1994. Using confidence intervals in within-subject designs. Psychon Bull Rev. 1:476-490.

Pashler H. 1988. Familiarity and visual change detection. Percept Psychophys. 44:369–378.

Worsley KJ. 2001. Statistical analysis of activation images. In: Jezzard P, Matthews PM, Smith SM, editors. Functional MRI: An introduction to methods. New York: Oxford University Press Inc. p. 251–270.
